# Minimax Nonparametric Parallelism Test

**Published:** 2020

**Authors:** Xin Xing, Meimei Liu, Ping Ma, Wenxuan Zhong

**Affiliations:** Department of Statistics, Virginia Tech, Blacksburg, VA, 24061, USA; Department of Statistics, University of Georgia, Athens, GA 30601, USA

**Keywords:** asymptotic distribution, minimax optimality, nonparametric inference, parallelism test, penalized least squares, smoothing spline ANOVA, Wald test

## Abstract

Testing the hypothesis of parallelism is a fundamental statistical problem arising from many applied sciences. In this paper, we develop a nonparametric parallelism test for inferring whether the trends are parallel in treatment and control groups. In particular, the proposed nonparametric parallelism test is a Wald type test based on a smoothing spline ANOVA (SSANOVA) model which can characterize the complex patterns of the data. We derive that the asymptotic null distribution of the test statistic is a Chi-square distribution, unveiling a new version of Wilks phenomenon. Notably, we establish the minimax sharp lower bound of the distinguishable rate for the nonparametric parallelism test by using the information theory, and further prove that the proposed test is minimax optimal. Simulation studies are conducted to investigate the empirical performance of the proposed test. DNA methylation and neuroimaging studies are presented to illustrate potential applications of the test. The software is available at https://github.com/BioAlgs/Parallelism.

## 1. Introduction

The assessment of parallelism is a fundamental problem in statistical inference and arises from many applications. For example, in genomic studies, one of primary interest is to detect genes with nonparallel expression patterns in time course studies (Storey et al., 2005; Ma et al., 2009). Another motivating example is in epigenomics, researchers are interested in testing whether the patterns of DNA methylation intensities along genome in the treatment and control groups are parallel or not (Hansen et al., 2012). The abnormal DNA methylation patterns are associated with changes in many important biological processes such as imprinting, X-chromosome inactivation, and aging (Schübeler, 2015). In functional neuroimaging, a common problem is to detect nonparallel signals (Nichols and Holmes, 2002; Orrison et al., 2017) among different brain regions.

There is an immense literature focused on the analysis of the parallelism of trends using linear model-based approaches, ranging from simple ANOVA (Sthle and Wold, 1989) to linear mixed models (Vossoughi et al., 2016). However, the linear model-based approaches have a limited ability to parsimoniously represent non-linear structures in complex data. Nonparametric parallelism comparison methods have drawn huge attention due to the modeling flexibility. Munk and Dette (1998) developed a test statistic through a weighted *L*_2_ distances for the regression functions based on similar equal-spaced fixed design. Degras et al. (2011) tested the parallelism of multiple time series based on the *L*_2_ distances between the local linear estimator of each individual curve and the global one for time series data when the time points are evenly spaced. Wang (1998) proposed a wavelet-based method to measure the changes of curves. Liu and Wang (2004) compared different nonparametric testing methods and showed that the performances of these tests depend on the shape of the true function. Ma et al. (2009) proposed an approximate F-test to detect nonparallel patterns in time course gene expression data with a more flexible random design.

However, rigorous testing methods with optimal power guarantees are still lacking in the existing nonparametric parallelism literature. The key cause of such research gap is that distinguishing from the simple/linear/polynomial null hypothesis, the parameter space of the null hypothesis for the nonparametric parallelism testing is a nonparametric function class with infinite dimension. How to conduct a rigorous test for such composite functional null hypothesis is still an open question. A major motivation of this article is on developing a nonparametric parallelism testing approach that detects the significance of the nonparallel effect, while guarantees statistical optimality in the sense of minimax testing rate, facilitating the power performance analysis.

In this article, we develop a nonparametric parallelism test based on the decomposition of tensor product reproducing Hilbert space (RKHS) (Wahba, 1990; Gu, 2013; Wang, 2011) under both fixed and random design. Tensor product RKHS provides a flexible space for modeling complex functions; see Wahba et al. (1995), Wood (2003) and reference therein. For the simplicity of description, we consider the case that there are two predictors only. Suppose the response variable *Y*_*ij*_ is the observed value of the *j*th subject at the *i*th time or spatial location for *i* = 1, ⋯ , *n* and *j* = 1, ⋯ , *s*. *Y*_*ij*_ depends on two predictors $x_{i}^{\langle 1\rangle }$ and $x_{j}^{\langle 2\rangle }$ through an unknown bivariate function f(⋅,⋅)∈H, the tensor product RKHS, where $x_{i}^{\langle 1\rangle }\in X_{1}=[0,1]$ is a continuous variable representing the *i*th time or the *i*-th spatial location, and $x_{j}^{\langle 2\rangle }\in X_{2}=\left\{0,1\right\}$ is a discrete variable representing the *j*th subject in different groups, $x_{j}^{\langle 2\rangle }=1$ represents the *j*th subject in treatment group, otherwise in control group. That is,

(1) $$Y_{ij}=f\left(x_{i}^{\langle 1\rangle },x_{j}^{\langle 2\rangle }\right)+\epsilon_{ij},i=1,\cdots n,j=1,\cdots ,s,$$

where *ϵ*_*ij*_s are i.i.d. random noise following a normal distribution with mean zero, variance *σ*^2^, and *s* is the number of subjects. Each subject can be represented by a curve. When *s* = 2, there are two curves in total and each group has only one curve. When *s* > 2, we have multiple curves in each group. We assume the i.i.d. random noise since, in many scientific experiments, the random errors are attributed to environmental factors independent of the time points or spatial location. For example, in the fMRI data analysis in Section 6, the error is mostly attributed to the random movement of the head and imaging noise which are independent with the time.

**Figure 1:**
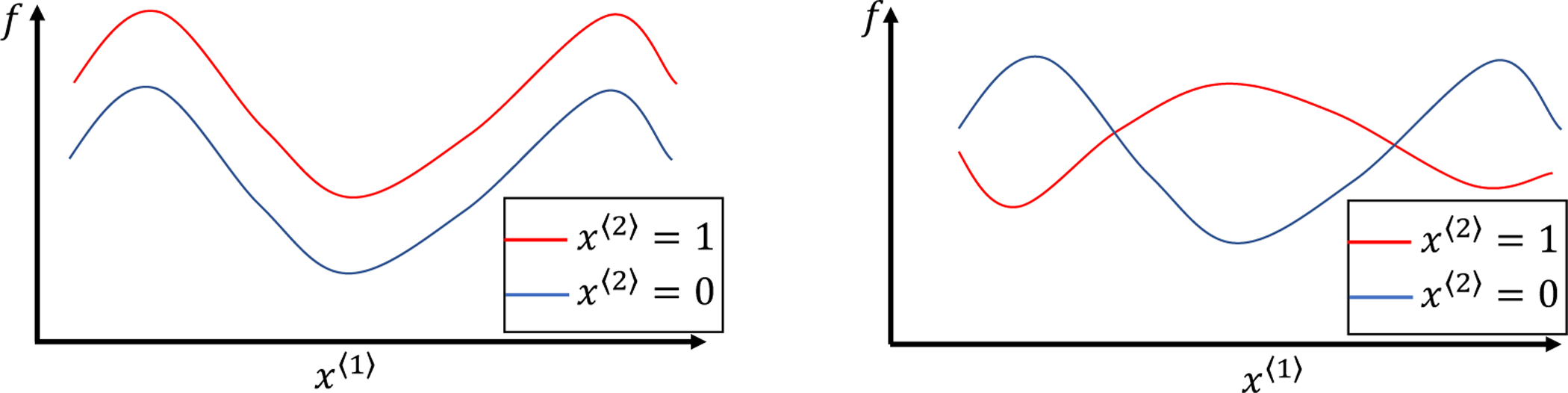
An illustration of two scenarios of a bivariate function *f*(*x*^〈1〉^, *x*^〈2〉^), where *x*^〈1〉^ is continuous, *x*^〈2〉^ only takes two values, 0 and 1. Left panel: the scenario with *f*_11_ = 0, i.e., *f*(*x*^〈1〉^, 0) and *f*(*x*^〈1〉^, 1) are parallel. Right panel: the scenario with *f*_11_ ≠ 0, i.e., *f*(*x*^〈1〉^, 0) and *f*(*x*^〈1〉^, 1) are nonparallel.

Analogous to the classical ANOVA decomposition, f∈H has the smoothing spline ANOVA (SSANOVA) decomposition (Wahba, 1990):

(2) $$f\left(x_{i}^{\langle 1\rangle },x_{j}^{\langle 2\rangle }\right)=f_{00}+f_{10}\left(x_{i}^{\langle 1\rangle }\right)+f_{01}\left(x_{j}^{\langle 2\rangle }\right)+f_{11}\left(x_{i}^{\langle 1\rangle },x_{j}^{\langle 2\rangle }\right),$$

where *f*_00_ is the grand mean, *f*_10_ and *f*_01_ are the main effects, and *f*_11_ is the nonparallel effect. When *f*_11_ = 0 (see the left panel in Figure 1), the curves in two groups are parallel. Then *f*_11_ = 0 is equivalent to that *f*(*x*^〈1〉^, 0) and *f*(*x*^〈1〉^, 1) are parallel. When *f*_11_ ≠ 0 (see the right panel in Figure 1), the magnitude of ${\left\|f_{11}\right\|}_{2}^{2}$ characterizes the significance of the non-parallelism between the treatment and control groups, where ${\left\|f_{11}\right\|}_{2}^{2}=\sum_{x^{\langle 2\rangle }=0}^{1}\int_{0}^{1}f^{2}\left(x^{\langle 1\rangle },x^{\langle 2\rangle }\right)d\omega_{1}$, with *ω*_1_ as the marginal density of *x*^〈1〉^. Statistically, the hypothesis testing for parallelism can be formulated as

(3) $$H_{0}:f_{11}=0\text{ vs }H_{1}:f_{11}\neq 0.$$

We introduce two concrete examples which motivate our study.

**Example 1.** *DNA methylation in case-control study*. DNA methylation is an essential epigenetic mechanism that regulates gene expression. Aberrant DNA methylation contributes to a number of human diseases including cancer (Stach et al., 2003). In a typical case-control study of DNA methylation (Filarsky et al., 2016), the DNA methylation level, denoted as *Y*_*ij*_ at the *i*th location $x_{i}^{\langle 1\rangle }$ on the genome for the *j*th individual in group $x_{j}^{\langle 2\rangle }$, can be modeled using Equation (1), where *f* is an unknown function with the SSANOVA decomposition in Equation (2). A primary focus is to infer whether the DNA methylation levels have different profiles along the genome between the case and control groups, i.e., testing the presence/absence of nonparallel effect *f*_11_ as in Equation (3).

**Example 2.** *Neuroimaging using functional magnetic resonance imaging (fMRI)*. fMRI is a powerful neuroimaging technology for the diagnosis of many brain-related diseases. It measures brain activity by detecting changes associated with blood flow. The primary form of fMRI uses the blood-oxygen-level dependent (BOLD) as signal (Huettel et al., 2004). In many case-control studies, the BOLD signal, *Y*_*ij*_, at the *i*th time $x_{i}^{\langle 1\rangle }$ for the *j*th subject in group $x_{j}^{\langle 2\rangle }$ is measured for a particular region of interest (ROI), and can be modeled using Equation (1), where *f* is an unknown function with the SSANOVA decomposition in Equation (2). The goal is to test whether the BOLD signals in two groups have same patterns along the time, i.e., test the significance of nonparallel effect *f*_11_ in Equation (3).

We first establish the minimax lower bound for nonparametric parallelism test in Equation (3) for general testing rules with the aid of tensor product decomposition of RKHS and the information theory. The tensor product decomposition in Equation (2) enables us to quantify the magnitude of nonparalelism by ∥*f*_11_∥_2_, where ∥ · ∥_2_ is the *L*_2_ norm. Intuitively, the smaller ∥*f*_11_∥_2_ is, the harder it is to distinguish the alternative hypothesis from the null. In analyzing the power performance, we consider a slightly different alternative hypothesis,

(4) $$H_{1}^{*}:{\left\|f_{11}\right\|}_{2}\geq d_{n},$$

where we remove the neighborhood within the *d*_*n*_ distance of *f*_11_ = 0 from the original alternative *H*_1_. Here the sequence *d*_*n*_ is called the distinguishable rate (or separation rate) (Ingster and Suslina, 2012; Giné and Nickl, 2015). We first introduce a geometric interpretation of the testing problem in Equation (3), and then establish a general minimax lower bound for the distinguishable rate for the nonparametric parallelism test using the Bernstein *k*-width in information theory (Pinkus, 2012). Bernstein *k*-width provides a geometric measure of the distinguishable rate and is easy to evaluate in the tensor product RKHS. Recently, similar technique was also used in analyzing the testing problems over cones and studied in Gaussian sequence models (Wei and Wainwright, 2020).

In addition, we propose a Wald-type test statistic as the squared empirical norm of the penalized least square estimator of *f*_11_. We derive its asymptotic null distribution, which satisfies the Wilks phenomenon. The asymptotic distribution of our test statistic is Gaussian, and the testing rule does not depend on any unknown quantities, thus is easy to compute. We can further reduce the computational cost by applying many popular fast computation methods such as fast random kernel methods Alaoui and Mahoney (2015) and subsampling methods such as Ma et al. (2015); Kim and Gu (2004). We note that our proposed Wald-type test distinguishes from the existing nonparametric testing methods as follows. The existing testing procedures mostly consider simple null hypothesis, such as the generalized likelihood ratio test in Fan et al. (2001), the penalized likelihood ratio test in Shang and Cheng (2013), the wavelet based method in Shen et al. (2002), and kernelized Stein method in Liu et al. (2016), whereas we consider a composite null hypothesis. More importantly, there is a nontrivial technical complication in addition to the above model setting difference. The composite null hypothesis *H*_0_ : *f*_11_ = 0 here defines a nonparametric function in an infinite-dimensional functional space rather than a parametric function in a finite-dimensional parameter space as required in Shang and Cheng (2013), because testing *H*_0_ : *f*_11_ = 0 is equivalent to testing *H*_0_ : *f* ∈ {*f*_00_ + *f*_10_ + *f*_01_}. Developing the limiting distribution of the test statistic in an infinite-dimensional null hypothesis space and quantifying the testing difficulty are very challenging since the distribution relies on the more delicate tensor product decomposition of the RKHS.

We further prove that the upper bound of the distinguishable rate for the proposed Wald type test matches the established minimax lower bound. Thus the proposed Wald-type test is minimax optimal. To the best of our knowledge, our work is the first one in establishing the minimax nonparametric parallelism test. Based on the Wald-type test statistic, we propose a data-adaptive choice of the regularization parameter with testing optimality guarantee.

The rest of the paper is organized as follows. We introduce the background of tensor product RKHS in Section 2. In Section 3, we introduce a minimax principle and a geometric interpretation of the parallelism testing problem. In Section 4, we derive the minimax lower bound of the distinguishable rate for general parallelism test using the information theory. Section 5 presents various simulation studies demonstrating substantial performance of our testing method, and Section 6 applies the methods to genome-wide anomaly of DNA methylation in *chronic lymphocytic leukemia* patients and brain function change in patients with *Alzheimer disease*. We conclude with a few remarks in Section 7. All technical proofs are relegated to the Appendix and Supplementary Material.

## 2. Background

In this section, we introduce some background of the tensor product RKHS, its tensor product decomposition, together with the penalized least square estimation.

### 2.1. Reproducing Kernel Hilbert Space

Given an RKHS H with an inner product ${\langle \cdot ,\cdot \rangle }_{H}$, there exists a symmetric and square integrable function K(⋅,⋅):X×X→ℝ such that

$${\langle f,K(x,\cdot )\rangle }_{H}=f(x),\text{ for all }f\in H\text{ and }x\in X.$$

We call K as the reproducing kernel of H. By Mercer’s theorem, any continuous kernel has the following decomposition

(5) $$K(x,y)=\overset{\infty }{\underset{\nu =0}{\sum }}\lambda_{\nu }\varphi_{\nu }(x)\varphi_{\nu }(y),$$

where *λ*_*ν*_s are non-negative descending eigenvalues and *φ*_*ν*_s are eigen-functions.

We consider the bivariate function *f* in Equation (1) on the product domain $X_{1}\times X_{2}$. We assume that *f* is a function in a tensor product RKHS (Lin, 2000)

(6) $$H=H^{(1\rangle }⊗H^{\langle 2\rangle }.$$

Given the Hilbert space $H^{\langle 1\rangle }$ and $H^{\langle 2\rangle }$, $H^{\langle 1\rangle }⊗H^{\langle 2\rangle }$ is defined as the completion of the class of functions with the form $\sum_{i=1}^{M}\eta_{1i}(x)\eta_{2i}(y)$, for $\eta_{1i}\in H^{\langle 1\rangle }$, $\eta_{2i}\in H^{\langle 2\rangle }$, and *M* is any positive integer. We consider $H^{\langle 1\rangle }$ as an *m*th order homogeneous Sobolev space, i.e.,

$$H^{\langle 1\rangle }=\{\eta_{1}\in L_{2}[0,1]|\eta_{1}^{\left(k\right)}\text{ is absolutely continuous and }\eta_{1}^{\left(k\right)}(0)=\eta_{1}^{\left(k\right)}(1)\text{ for }k=0,1,\ldots ,m-1,\eta_{1}^{\left(m\right)}\in L_{2}[0,1]\},$$

and $H^{\langle 2\rangle }$ is a two-dimensional Euclidean space with standard Euclidean norm.

Assume that $H^{\langle 1\rangle }$ has the eigenvalue and eigenvector pairs ${\left\{\mu_{i},\phi_{i}\right\}}_{i=0}^{\infty }$ and $H^{\langle 2\rangle }$ has the eigenvalue and eigenvector pairs ${\left\{\nu_{j},\psi_{j}\right\}}_{j=1}^{2}$. Then we have the eigenvalue and eigenvector pairs for the kernel function K in H as

(7) $$\left\{\mu_{i}\nu_{j},\phi_{i}\psi_{j}\right\}\text{ for }i=0,\ldots ,\infty ,j=1,2,$$

in the decomposition in Equation (5). We refer Equation (7) as the eigensystem for H. We further denote ${\langle \cdot ,\cdot \rangle }_{H}$ as the product norm induced by the norm on the marginal space $H_{1}$ and $H_{2}$ (Lin, 2000).

Using the Riesz representation theorem (Schölkopf et al., 2001), we can easily represent any function f∈H as in the following Lemma.

**Lemma 1** *Given the sampling points* $x_{ij}=\left(x_{i}^{\langle 1\rangle },x_{j}^{\langle 2\rangle }\right)$, *i* = 1, ⋯ , *n and j* = 1, ⋯ *s*, *for any f in a reproducing kernel Hilbert space* H, *there exists a set of reproducing kernels* $K_{x_{ij}}(\cdot ,\cdot )$ *such that*

(8) $$f\left(x^{\langle 1\rangle },x^{\langle 2\rangle }\right)=\overset{n}{\underset{i=1}{\sum }}\overset{s}{\underset{j=1}{\sum }}\alpha_{ij}K_{x_{ij}}\left(x^{\langle 1\rangle },x^{\langle 2\rangle }\right)+\rho \left(x^{\langle 1\rangle },x^{\langle 2\rangle }\right).$$

Lemma 1 implies that *f* can be expressed as a sum of a linear expansion of $K_{x_{ij}}$ and a nonlinear function *ρ*. Notice that when $\left(x^{\langle 1\rangle },x^{\langle 2\rangle }\right)\in {\left\{x_{ij}\right\}}_{i=1,\cdots ,n}^{j=1,\cdots ,s}$, we have *ρ*(*x*^〈1〉^, *x*^〈2〉^) = 0. Thus, *ρ*(·,·) can be considered as a residual that quantifies the unknown information of function *f*. To get an estimate of *f*, we only need to specify $K_{x_{ij}}(\cdot ,\cdot )$ and estimate *α*_*ij*_. Next, we provide a way to construct the reproducing kernels $K_{x_{ij}}(\cdot ,\cdot )$. In order to do that, we need the following two lemmas.

**Lemma 2** *Suppose* $K^{\langle 1\rangle }$ *is the reproducing kernel of* $H^{\langle 1\rangle }$ *on* $X_{1}$, *and* $K^{\langle 2\rangle }$ *is the reproducing kernel of* $H^{\langle 2\rangle }$ *on* $X_{2}$. *Then the reproducing kernels of* $H^{\langle 1\rangle }⊗H^{\langle 2\rangle }$ *on* $X=X_{1}\times X_{2}$ *is* $K(x,z)=K^{\langle 1\rangle }\left(x^{\langle 1\rangle },z^{\langle 1\rangle }\right)K^{\langle 2\rangle }\left(x^{\langle 2\rangle },z^{\langle 2\rangle }\right)$ *with* **x** = (*x*^〈1〉^, *x*^〈2〉^) *and* **z** = (*z*^〈1〉^, *z*^〈2〉^.

**Lemma 3** *For every Sobolev space* H *of functions on* X, *there corresponds a unique reproducing kernel* K, *which is non-negative definite*. *If* $K_{0}$ *and* $K_{1}$ *are both non-negative definite reproducing kernels for* $H_{0}$ *and* $H_{1}$, *and* $H_{0}\cap H_{1}=\left\{0\right\}$, *then* $H_{0}⊕H_{1}$ *has a reproducing kernel* $K=K_{0}+K_{1}$.

Lemmas 2 and 3 can be easily proved based on Theorems 2.3 to 2.6 in Gu (2013). Lemma 2 states that the reproducing kernel of the tensor product space is the product of the reproducing kernels. Lemma 3 states that the reproducing kernel of a tensor sum space is the sum of the reproducing kernels. Therefore, to construct $K_{x_{ij}}(\cdot ,\cdot )$, we introduce the decomposition of tensor product space in the following part.

### 2.2. Decomposition of Tensor Product Space

For any $\eta_{1}\in H^{\langle 1\rangle }$ and $\eta_{2}\in H^{\langle 2\rangle }$, define the averaging operators $A_{1}:\eta_{1}\to \int_{0}^{1}\eta_{1}(x)dx$ and $A_{2}:\eta_{2}\to \frac{1}{2}\sum_{k=1}^{2}\eta_{2}(k)$ where $\eta_{2}(k)=e_{k}^{T}\eta_{2}$, *e*_*k*_ is the unit vector with the *k*th element one and all other elements zeros. We have $H^{\langle 1\rangle }$ and $H^{\langle 2\rangle }$ with the following tensor sum decomposition $H_{0}^{\langle 1\rangle }⊕H_{1}^{\langle 1\rangle }$ and $H_{0}^{\langle 2\rangle }⊕H_{1}^{\langle 2\rangle }$ respectively, where $H_{0}^{\langle 1\rangle }=\left\{A_{1}\eta_{1}|\eta_{1}\in H^{\langle 1\rangle }\right\}$, $H_{0}^{\langle 2\rangle }=\left\{A_{2}\eta_{2}|\eta_{2}\in H^{\langle 2\rangle }\right\}$, $H_{1}^{\langle 1\rangle }=\left\{\left(I-A_{1}\right)\eta_{1}|\eta_{1}\in H^{\langle 1\rangle }\right\}$, $H_{1}^{\langle 2\rangle }=\left\{\left(I-A_{2}\right)\eta_{2}|\eta_{2}\in {\mathbb{R}}^{2}\right\}$, and I is the operator. Thus H has the following tensor sum decomposition

(9) $$H=\left(H_{0}^{\langle 1\rangle }⊗H_{0}^{\langle 2\rangle }\right)⊕\left(H_{1}^{\langle 1\rangle }⊗H_{0}^{(2\rangle }\right)⊕\left(H_{0}^{\langle 1\rangle }⊗H_{1}^{\langle 2\rangle }\right)⊕\left(H_{1}^{\langle 1\rangle }⊗H_{1}^{\langle 2\rangle }\right),$$

and for any $f\in H^{\langle 1\rangle }⊗H^{\langle 2\rangle }$, we have

(10) $$f=f_{00}+f_{10}+f_{01}+f_{11},$$

where $f_{00}=A_{1}A_{2}f\in H_{0}^{\langle 1\rangle }⊗H_{0}^{\langle 2\rangle }$, $f_{10}=\left(I-A_{1}\right)A_{2}f\in H_{1}^{\langle 1\rangle }⊗H_{0}^{\langle 2\rangle }$, $f_{01}=A_{1}\left(I-A_{2}\right)f\in H_{0}^{1}⊗H_{1}^{2}$ and $f_{11}=\left(I-A_{1}\right)\left(I-A_{2}\right)f\in H_{1}^{\langle 1\rangle }⊗H_{1}^{\langle 2\rangle }$. Thus, any function f∈H can be decomposed uniquely as : *f*_00_ the interception, *f*_10_ and *f*_01_ the marginal effects and *f*_11_ the two-way interaction term.

Denote the reproducing kernels of $H_{0}^{\langle 1\rangle }$, $H_{0}^{\langle 2\rangle }$, $H_{1}^{\langle 1\rangle }$, $H_{1}^{\langle 2\rangle }$ as $K_{0}^{\langle 1\rangle }$, $K_{1}^{\langle 1\rangle }$, $K_{0}^{\langle 2\rangle }$, $K_{1}^{\langle 2\rangle }$, respectively. Specifically, $K_{0}^{\langle 1\rangle }\left(x^{\langle 1\rangle },z^{\langle 1\rangle }\right)=1$ and $K_{1}^{\langle 1\rangle }\left(x^{\langle 1\rangle },z^{\langle 1\rangle }\right)$ is defined as (−1)^*m*−1^*k*_2*m*_(*z*^〈1〉^ − *x*^〈1〉^) for the *m*th order homogeneous subspace where *k*_*r*_(·) is the *r*th order scaled Bernoulli polynomials (Abramowitz and Stegun, 1964; Gu, 2013) and **1**_(·)_ is the indicator function. $K_{0}^{\langle 2\rangle }\left(x^{\langle 2\rangle },z^{\langle 2\rangle }\right)=1/2$ and $K_{1}^{\langle 2\rangle }\left(x^{\langle 2\rangle },z^{\langle 2\rangle }\right)=1_{\left(z^{\langle 2\rangle }=x^{\langle 2\rangle }\right)}-1/2$ on $X_{2}$. Let $H_{ll^{\prime }}=H_{l}^{\langle 1\rangle }⊗H_{l^{\prime }}^{\langle 2\rangle }$ with reproducing kernel $K^{ll^{\prime }}$, where

$$K^{ll^{\prime }}\left(x_{ij},x_{i^{\prime }j^{\prime }}\right)=K_{l}^{\langle 1\rangle }\left(x_{i}^{\langle 1\rangle },x_{i^{\prime }}^{\langle 1\rangle }\right)K_{l^{\prime }}^{\langle 2\rangle }\left(x_{j^{\prime }}^{\langle 2\rangle },x_{j^{\prime }}^{\langle 2\rangle }\right),$$

for *ℓ*,*ℓ*′ ∈ {0, 1}. The induced inner product of $H_{ll^{\prime }}$ is denoted as 〈*f*_*ℓℓ*′_, *g*_*ℓℓ*′_〉_*ℓℓ*′_, where *f*_*ℓℓ*′_ and *g*_*ℓℓ*′_ are projections of *f* and *g* on $H_{ll^{\prime }}$ respectively, *ℓ*,*ℓ*′ ∈ {0, 1}. Notice that the metrics induced by inner products 〈*f*_*ℓℓ*′_, *g*_*ℓℓ*′_〉_*ℓℓ*′_ are not necessarily of the same scale for different *ℓℓ*^′^. The inner product for H can be defined as

(11) $${\langle f,g\rangle }_{H}=\underset{ll^{\prime }}{\sum }\theta_{ll^{\prime }}^{-1}{\langle f_{ll^{\prime }},g_{ll^{\prime }}\rangle }_{ll^{\prime }},$$

where *θ*_*ℓℓ*′_s re-scale the metrics on different $H_{ll^{\prime }}$, 〈·,·〉_*ℓℓ*′_ is the restricted norm of ${\langle \cdot ,\cdot \rangle }_{H}$ on $H_{ll^{\prime }}$.

Based on Lemmas 2 and 3, we can easily show that the reproducing kernels associated with Equation (11) is $K\left(x_{ij},x_{i^{\prime }j^{\prime }}\right)=\sum_{l,l^{\prime }}\theta_{ll^{\prime }}K^{ll^{\prime }}\left(x_{ij},x_{i^{\prime }j^{\prime }}\right)$ with *ℓ*,*ℓ*′ = 0, 1. Thus, given the sampling points $x_{ij}=\left(x_{i}^{\langle 1\rangle },x_{j}^{\langle 2\rangle }\right)$ for *i* = 1, ⋯ , *n* and *j* = 1, ⋯ , *s*, the kernel function in H is a bivariate function depending on **x**_*ij*_, i.e.,

(12) $$K_{x_{ij}}\left(x^{\langle 1\rangle },x^{\langle 2\rangle }\right)=\frac{\theta_{00}}{2}+\theta_{01}\left(1_{\left(x^{\langle 2\rangle }=x_{j}^{\langle 2\rangle }\right)}-\frac{1}{2}\right)+\theta_{10}\frac{1}{2}K_{1}^{\langle 1\rangle }\left(x^{\langle 1\rangle },z^{\langle 1\rangle }\right)+\theta_{11}\frac{1}{2}\left(1_{\left(x^{\langle 2\rangle }=x_{j}^{\langle 2\rangle }\right)}-\frac{1}{2}\right)K_{1}^{\langle 1\rangle }\left(x^{\langle 1\rangle },z^{\langle 1\rangle }\right),$$

and accordingly $f\left(x^{\langle 1\rangle },x^{\langle 2\rangle }\right)=\sum_{ij}\alpha_{ij}K_{x_{ij}}\left(x^{\langle 1\rangle },x^{\langle 2\rangle }\right)+\rho \left(x^{\langle 1\rangle },x^{\langle 2\rangle }\right)$ by Lemma 1.

In the function decomposition in Equation (10), it is easy to verify that $f_{00}\in H_{00}=\left\{g:g=\left\{\left(\theta_{00}-\theta_{01}\right)/2\right\}\sum_{ij}\alpha_{ij}\right\}$. As *f*_00_ is a constant for any *x*^〈1〉^ and *x*^〈2〉^, it is analogous to the ground mean in classical ANOVA models. Similarly, we have $f_{01}\in H_{01}=\left\{g:g=\theta_{01}\sum_{ij}\alpha_{ij}1_{\left(x^{\langle 2\rangle }=x_{j}^{\langle 2\rangle }\right)}\right\}$. Recall that $x_{j}^{\langle 2\rangle }$ can only be either 0 or 1, we can rewrite *f*_01_ as $1_{\left(x^{\langle 2\rangle }=0\right)}\beta_{0}+1_{\left(x^{\langle 2\rangle }=1\right)}\beta_{1}$, where $\beta_{0}=\sum_{j=1}^{s}\left(\sum_{i=1}^{n}\alpha_{ij}\right)1_{\left(x_{j}^{\langle 2\rangle }=0\right)}$ and $\beta_{1}=\sum_{j=1}^{s}\left(\sum_{i=1}^{n}\alpha_{ij}\right)1_{\left(x_{j}^{\langle 2\rangle }=1\right)}$.

We remark that *f*_00_ and *f*_01_ are all in a finite-dimensional space. The space $H_{10}$ (where *f*_10_ lies in) spanned by the third term in the right hand side of Equation (12) is, however, an infinite-dimensional space, because we have uncountable $x\in X_{1}$. The function can be expressed as a linear combination of the observed reproducing kernels plus a residual that quantifies the unobserved reproducing kernels, i.e., $H_{10}=\left\{g:g=\frac{1}{2}\sum_{i=1}^{n}\left(\theta_{10}\sum_{j=1}^{s}\alpha_{ij}\right)K_{1}^{\langle 1\rangle }\left(x^{\langle 1\rangle },z^{\langle 1\rangle }\right)+\rho_{2}\right\}$. Notice that function in this space only changes as we change *x*^〈1〉^. Thus, the third term in right hand side of (12) can be used to quantify the effect of the continuous variable such as the temporal effect. The forth term in the right hand side of Equation (12) varies for both continuous variable and the case-control indicator, thus it is the term that can catch different functional patterns between the case and control. Similarly, the space spanned by the last addend is also an infinite-dimensional space because we still have an infinite number of unobserved kernel functions in addition to the *n* × *s* observed kernel functions. Thus, we he $f_{11}\in H_{11}=\left\{g:g=\frac{1}{2}\theta_{11}\sum_{ij}\alpha_{ij}\left(1_{\left(x_{j}^{\langle 2\rangle }=x^{2}\right)}-\frac{1}{2}\right)K_{1}^{\langle 1\rangle }\left(x^{\langle 1\rangle },z^{\langle 1\rangle }\right)+\rho_{12}\right\}$. Clearly, to test if two functions are parallel to each other, we only need to test if *f*_11_ = 0.

### 2.3. Penalized Least Squares

Here we introduce the penalized least square estimate of f∈H, and the interaction term *f*_11_ in Equation (10). Given the sampling points $x_{ij}=\left(x_{i}^{\langle 1\rangle },x_{j}^{\langle 2\rangle }\right)$ for *i* = 1, …, *n* and *j* = 1, …, *s*, consider the model space

$$H_{\text{model}}=\left\{g:g=\overset{n}{\underset{i=1}{\sum }}\overset{s}{\underset{j=1}{\sum }}\alpha_{ij}K_{x_{ij}}\left(x^{\langle 1\rangle },x^{\langle 2\rangle }\right)\right\},$$

a closed linear subspace of H. *α*_*ij*_s are the regression coefficients, and the bivariate residual function *ρ*(·,·) in Lemma 1 is in $H_{\text{residual}}=H⊖H_{\text{model}}$. Notice that $\rho \left(x_{i}^{\langle 1\rangle },x_{j}^{\langle 2\rangle }\right)=\langle K_{x_{ij}}\left(x^{\langle 1\rangle },x^{\langle 2\rangle }\right),\rho \rangle =0$ because of the orthogonality constraint between $H_{\text{model}}$ and $H_{\text{residual}}$. Then, *f* can be estimated by minimizing the penalized least squares functional as follows:

(13) $$\frac{1}{ns}\overset{n}{\underset{i=1}{\sum }}\overset{s}{\underset{j=1}{\sum }}{\left(Y_{ij}-\underset{i^{\prime }j^{\prime }}{\sum }\alpha_{i^{\prime }j^{\prime }}K_{x_{i^{\prime }j^{\prime }}}\left(x_{i}^{\langle 1\rangle },x_{j}^{\langle 2\rangle }\right)\right)}^{2}+\lambda J\left(f_{10}+f_{11}\right),$$

where the quadratic functional $J(f)=J\left(f_{10}+f_{11}\right)={\left\|f_{10}+f_{11}\right\|}_{H}^{2}$ quantifies the roughness of *f*_10_ and *f*_11_, the smoothing parameter *λ* controls the trade-off between the goodness-of-fit and the roughness of *f*_10_ and *f*_11_. Recall *ρ* and $K_{x_{i^{\prime }j^{\prime }}}(\cdot ,\cdot )$ are orthogonal to each other. Plugging Equation (8) into *J*(*f*), we have

$$J(f)={\langle \sum_{i^{\prime }j^{\prime }}\alpha_{i^{\prime }j^{\prime }}\left(\theta_{10}K_{x_{i^{\prime }j^{\prime }}}^{10}+\theta_{11}K_{x_{i^{\prime }j^{\prime }}}^{11}\right),\sum_{i^{\prime }j^{\prime }}\alpha_{i^{\prime }j^{\prime }}\left(\theta_{10}K_{x_{i^{\prime }j^{\prime }}}^{10}+\theta_{11}K_{x_{i^{\prime }j^{\prime }}}^{11}\right)\rangle }_{H}+{\langle \rho ,\rho \rangle }_{H}.$$

Further notice that $\langle K_{x_{ij}}^{ll^{\prime }},K_{x_{i^{\prime }j^{\prime }}}^{ll^{\prime }}\rangle =K_{x_{ij}}^{ll^{\prime }}\left(x_{i^{\prime }}^{\langle 1\rangle },x_{j^{\prime }}^{\langle 2\rangle }\right)$ by the reproducible property of reproducing kernels (Gu, 2013). Thus, substituting K and $K^{ll^{\prime }}$ by (12) and *f* in *J*(*f*) by Equation (8), Equation (13) can be rewritten as

(14) $$\|y-\text{nsK}\alpha {\|}_{2}^{2}+ns\lambda \alpha^{T}Q\alpha +ns\lambda {\langle \rho ,\rho \rangle }_{H},$$

where **y** = (*Y*_11_, *Y*_21_, …, *Y*_*ns*_)^*T*^, *K* is the *ns* × *ns* matrix with (*i* + *n*(*j* − 1), *i*′ + *n*(*j*′ − 1))th entry $\frac{1}{ns}K_{x_{ij}}\left(x_{i^{\prime }}^{\langle 1\rangle },x_{j^{\prime }}^{\langle 2\rangle }\right)$, *Q* is the *ns* × *ns* matrix with (*i* + *n*(*j* − 1), *i*′ + *n*(*j*′ − 1))th entry $\frac{1}{ns}\left(\theta^{10}K_{x_{ij}}^{10}\left(x_{i^{\prime }}^{\langle 1\rangle },x_{j^{\prime }}^{\langle 2\rangle }\right)+\theta^{11}K_{x_{ij}}^{11}\left(x_{i^{\prime }}^{\langle 1\rangle },x_{j^{\prime }}^{\langle 2\rangle }\right)\right)$ and ***α*** = (*α*_11_, *α*_21_, …, *α*_*ns*_)^*T*^. Similar to Chapter A3 in Gu (2013), we set the rescale parameter *θ*_10_ and *θ*_11_ to make $\theta_{10}K^{10}$ and $\theta_{11}K^{11}$ contribute equally in penalty term of Equation (14) (see Appendix A.1 for details) and set *θ*_00_ and *θ*_01_ as one since $H_{00}$ and $H_{01}$ are simply one-dimensional Euclidean space. Since *ρ* does not rely on *α*, the optimizer of ***α*** in minimizing Equation (14) is equivalent to minimizing

(15) $$\hat{\alpha }=\underset{\alpha \in {\mathbb{R}}^{ns}}{\text{arg min}}\|y-\text{nsK}\alpha {\|}_{2}^{2}+ns\lambda \alpha^{T}Q\alpha .$$

The penalized least square estimate of *f* is then $\hat{f}\left(x_{i}^{\langle 1\rangle },x_{j}^{\langle 2\rangle }\right)=\sum_{i,j}^{n,s}{\hat{\alpha }}_{i,j}K_{x_{i,j}}\left(x_{i}^{\langle 1\rangle },x_{j}^{\langle 2\rangle }\right)$.

As *n* goes to infinity, we have countable number of kernels and *f*(*x*^〈1〉^, *x*^〈2〉^) that the minimizer of Equation (13) resides in an infinite dimensional space spanned by a countable number of kernels, i.e.,

$$H_{\text{model}}^{\infty }=\left\{g:g\left(x^{\langle 1\rangle },x^{\langle 2\rangle }\right)=\overset{\infty }{\underset{ij}{\sum }}\alpha_{ij}K_{x_{ij}}\left(x^{\langle 1\rangle },x^{\langle 2\rangle }\right)\right\}.$$

The nonparallel effect *f*_11_ also resides in a subspace that is spanned by a countable number of kernels. We denote the subspace by

$$H_{11}^{\infty }=\left\{f_{11}:f_{11}\left(x^{\langle 1\rangle },x^{\langle 2\rangle }\right)=\overset{\infty }{\underset{ij}{\sum }}\alpha_{ij}\frac{{\left(-1\right)}^{m-1}}{2}\left(1_{\left(x_{j}^{\left(2\right)}=x^{\left(2\right)}\right)}-\frac{1}{2}\right)k_{2m}\left(x_{i}^{\langle 1\rangle }-x^{\langle 1\rangle }\right)\right\}.$$

Here, we did not normalize *f*_11_ by the constant scale parameter *θ*_11_ for the simplicity of description. The penalized least square estimate of $f_{11}\in H_{11}^{\infty }$ is

(16) $${\hat{f}}_{11}\left(x^{\langle 1\rangle },x^{\langle 2\rangle }\right)=\overset{n,s}{\underset{i,j}{\sum }}{\hat{\alpha }}_{ij}\frac{{\left(-1\right)}^{m-1}}{2}\left(1_{\left(x_{j}^{\langle 2\rangle }=x^{\langle 2\rangle }\right)}-\frac{1}{2}\right)k_{2m}\left(x_{i}^{\langle 1\rangle }-x^{\langle 1\rangle }\right).$$

With a little abuse of notation, we use ${\hat{f}}_{11}$ to denote the vector version evaluation of ${\hat{f}}_{11}$ on *ns* data points from now on. Plugging in $\hat{\alpha }$ to (16), we have an explicit expression of ${\hat{f}}_{11}$ as

(17) $${\hat{f}}_{11}=K_{11}M^{-1}\left(I_{ns}-S{\left(S^{T}M^{-1}S\right)}^{-1}S^{T}M^{-1}\right)y,$$

where *I*_*ns*_ is the *ns* dimensional identity matrix, *S*, *M* and *K*_11_ are reparametrization of the kernel matrices with explicit forms provided in Appendix A.1–“Notation Clarification”.

In Section 4, we will construct a Wald type test statistics based on ${\hat{f}}_{11}$ for the parallelism test *H*_0_ : *f*_11_ = 0, and derive its null asymptotic distribution. Before that, we first establish the minimax principle of the parallelism test for general testing rules in the following Section 3.

## 3. Minimax Principle of the Nonparametric Parallelism Test

Consider the test problem as follows

(18) $$H_{0}:f_{11}=0\;vs\;H_{1}:{\left\|f_{11}\right\|}_{2}>0.$$

Given a decision rule *ϕ*_*n*_ for the testing problem (18), *ϕ*_*n*_ = 0 if *H*_0_ is preferred and 1 otherwise. Then the zero-one loss function is

(19) $$\text{Loss}\left(\phi_{n}\right)=\{\begin{array}{ll} \phi_{n} & \text{ if }H_{0}\text{ is true},\\ 1-\phi_{n} & \text{ if }H_{1}\text{ is true}. \end{array}$$

The minimax principle requires *ϕ*_*n*_ to minimize the maximum possible risk, i.e.,

(20) $$\underset{\phi_{n}}{\text{min }}\underset{H}{\text{max }}E\left[\text{Loss}\left(\phi_{n}\right)\right]=\underset{\phi_{n}}{\text{min}}\left[\underset{H_{0}}{\text{max }}E\left(\phi_{n}|H_{0}\text{ is true}\right)+\underset{H_{1}}{\text{max }}E\left(1-\phi_{n}|H_{1}\text{ is true}\right)\right].$$

Notice $E\left(\phi_{n}|H_{0}\text{ is true}\right)$ is the probability of making a type I error and $E\left(1-\phi_{n}|H_{1}\text{ is true}\right)$ is the probability of making a type II error. Intuitively, we choose *ϕ*_*n*_ to minimize the maximum possible type I error and type II error. Notice that if *H*_0_ and *H*_1_ are contiguous, we cannot ensure that Equation (20) can be controlled, because there may lie some *f*_11_ on the boundary of *H*_0_ and *H*_1_ for which strikes the balance between acceptance and rejection of the null hypothesis, and an appropriate decision cannot be made. Thus, instead of *H*_1_, we consider a slightly different alternative hypothesis (4) and partition the parameter space into three sets: $H_{0}+H_{1}^{*}+I$, of which *I* designates the indifference zone 0 < ∥*f*_11_∥_2_ < *d*_*n*_. Because *d*_*n*_ clearly separates *H*_0_ from $H_{1}^{*}$, it is referred to as the distinguishable rate (a.k.a the separation rate) (Ingster and Suslina, 2012; Giné and Nickl, 2015). Let

(21) $$\text{pseudo.risk}\left(\phi_{n},d_{n}\right)=\underset{H_{0}}{\text{sup }}E\left(\phi_{n}|H_{0}\text{ is true}\right)+\underset{H_{1}^{*}}{\text{sup }}E\left(1-\phi_{n}|H_{1}^{*}\text{ is true}\right).$$

Then pseudo.risk(*ϕ*_*n*_, *d*_*n*_) converges to the risk function $E\left[\text{Loss}\left(\phi_{n}\right)\right]$ as *d*_*n*_ goes to zero.

Compared to the risk function, the pseudo.risk is not only a function of a decision rule *ϕ*_*n*_ but also a function of the distinguishable rate *d*_*n*_. When *ϕ*_*n*_ is given, we have ${\text{sup}}_{H_{1}^{*}}\;E\left(1-\phi_{n}|H_{1}^{*}\text{ is true}\right)\leq {\text{sup}}_{H_{1}}\;E\left(1-\phi_{n}|H_{1}\text{ is true}\right)$ because $H_{1}^{*}$ is a subset of *H*_1_. Thus, finding the largest pseudo.risk on $H_{1}^{*}$ for a given *ϕ*_*n*_ is equivalent to finding the smallest *d*_*n*_ with a tolerable pseudo.risk. In another word, finding the maximum possible pseudo.risk over the parameter space can be considered as finding the smallest boundary of $H_{1}^{*}$ such that an appropriate decision *ϕ*_*n*_ can be made and the risk can be controlled. Meanwhile, for an adequately large given *d*_*n*_, we can always find a decision rule such that the pseudo.risk can reach its minimum value. Let $\phi_{n}^{†}\left(d_{n}\right)=\text{arg }{\text{min}}_{\phi_{n}}$ pseudo.risk(*ϕ*_*n*_, *d*_*n*_). Then, if *d*_*n*_ can reach the smallest value $d_{n}^{†}$, the corresponding $\phi_{n}^{†}\left(d_{n}^{†}\right)$ is the minimax decision. Thus, the essential step to find the minimax decision of pseudo.risk(*ϕ*_*n*_, *d*_*n*_) is to find $d_{n}^{†}$ such that

(22) $$d_{n}^{†}=\underset{d_{n}}{\text{arg min }}\phi_{n}^{†}\left(d_{n}\right).$$

Because $d_{n}^{†}$ is an estimate of the distinguishable rate to obtain the minimax test, it is referred to as the minimax distinguishable rate. Clearly, the corresponding decision rule $\phi_{n}^{†}$ is the minimax decision rule.

**Figure 2:**
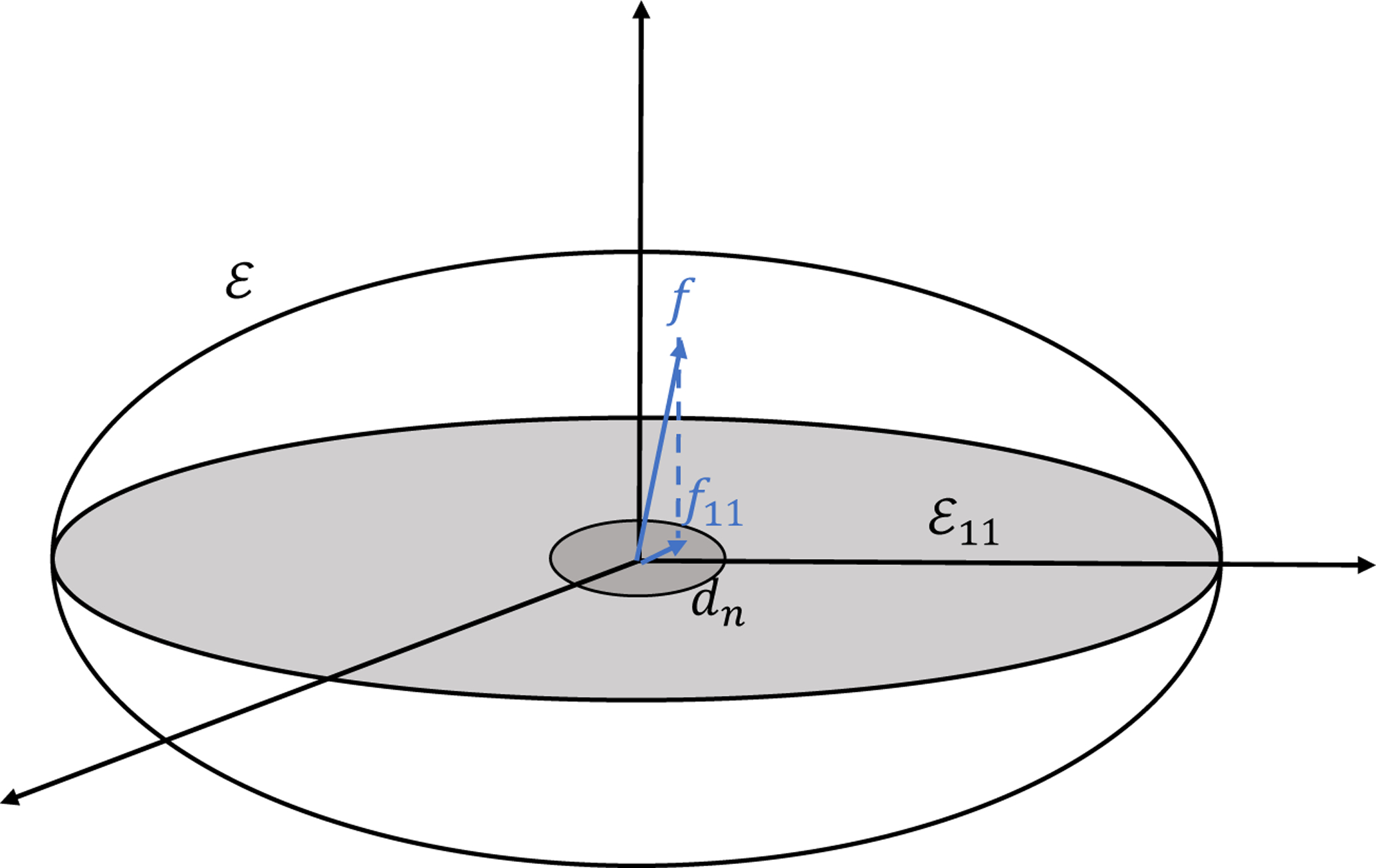
Geometric interpretation of the distinguishable rate of the parallelism test.

We first introduce a geometric interpretation of the testing problem (18). Geometrically, we can treat $E=\left\{f\in H:\|f{\|}_{H}<1/2\right\}$ as an ellipse with eigenvalues in Equation (7) as axis lengths as shown in Figure 2. For any f∈E, the projection of *f* on $\left\{f:f\in E_{11}:=H_{11}\cap E\right\}$ is *f*_11_. The magnitude of nonparallelism can be qualified by ∥*f*_11_∥_2_. The distinguishable rate *d*_*n*_ is the radius of the sphere centered at *f*_11_ = 0 in $H_{11}$.

Intuitively, the testing will be harder when the projection of *f* on $E_{11}$ is closer to the origin *f*_11_ = 0. We use the Bernstern width in Pinkus (2012) to characterize the testing difficulty. Let *S*_*k*+1_ be the set of all (*k* + 1)-dimensional subspaces for any *k* ≥ 1. For a compact set *C*, the Bernstein *k*-width is defined as

(23) $$b_{k,2}(C)≔\underset{r\geq 0}{\text{arg max}}\left\{B_{2}^{k+1}(r)\subset C\cap S\text{ for some subspace }S\in S_{k+1}\right\},$$

where $B_{2}^{k+1}(r)$ is a (*k* + 1)-dimensional *l*_2_-ball with radius *r* centered at *f*_11_ = 0 in $E_{11}$. The Bernstein width characterizes the largest ball that can be inserted into a (*k*+1)-dimensional subspace in $E_{11}$. Based on the Bernstein width, we give an upper bound of the testing radius, i.e., for any *f* projected in the ball with radius less than this upper bound, the minimum pseudo.risk is larger than 1/2.

**Lemma 4** *For any* f∈H, *we have*

$$\underset{\phi_{n}}{\text{inf}}\text{ pseudo.risk}\left(\phi_{n},d_{n}\right)\geq 1/2$$

*for all*

$$d_{n}\leq r_{B}≔\text{sup}\left\{\delta |\delta \leq \frac{1}{2\sqrt{n}}\sigma {\left(k_{B}(\delta )\right)}^{1/4}\right\}$$

*where* $k_{B}(\delta )≔\text{arg }{\text{max}}_{k}\left\{b_{k-1,2}^{2}\left(H_{11}\right)\geq \delta^{2}\right\}$ *is the Bernstein lower critical dimension, and r*_*B*_ *is called the Bernstein lower critical radius*.

Lemma 4 shows that when *d*_*n*_ is less than *r*_*B*_, there has no test can distinguish the alternative hypothesis from the null. In order to achieve a non-trivial power, we need *d*_*n*_ to be larger than the Bernstein lower critical radius *r*_*B*_, which is determined by the Bernstein lower critical dimension *k*_*B*_(*δ*). In the next lemma, we provide the lower bound for *k*_*B*_(*δ*).

**Lemma 5** *Let* ${\left\{\rho_{i}\right\}}_{i=1}^{\infty }$ *be eigenvalues of* $H_{11}$. *We have*

(24) $$k_{B}(\delta )>\underset{k}{\text{arg max}}\left\{\sqrt{\rho_{k}}\geq \delta \right\}$$

Plugging in the lower bound of *k*_*B*_(*δ*) derived in Lemma 5 to Lemma 4, we calculate a lower bound for *r*_*B*_ based on the decay rate of eigenvalues. *r*_*B*_ is served as a minimax lower bound for the distinguishable rate. The following theorem summarizes the minimax distinguishable rate for the testing problem (18).

**Theorem 6 *(Minimax lower bound for distinguishable rate)*** *In the nonparametric model (1) with SSANOVA (2). Suppose* f∈H, *where* $H=H^{\langle 1\rangle }⊗H^{\langle 2\rangle }$ *with* $H^{\langle 1\rangle }$ *as the mth order Sobolev space*^1^, *and* $H^{\langle 2\rangle }$ *as a two-dimensional Euclidean space. The minimax distinguishable rate for testing hypotheses* (18) *is achieved at* $d_{n}^{†}≳n^{-2m/(4m+1)}$.

Theorem 6 provides a general guidance for justifying a local minimax test, i.e., there is no test can distinguish the alternative from null if *d*_*n*_ ≲ *n*^−2*m*/(4*m*+1)^. The proof of Theorem 6 is presented in the Appendix. Essentially, for any test *ϕ*_*n*_ that is defined by a family of type I error $\alpha =E\left(\phi_{n}\right)$ and by the supremum of the type II error $\delta ={\text{sup}}_{H_{1}^{*}}\;E\left(1-\phi_{n}|H_{1}^{*}\text{ is true}\right)$, we need *ϕ*_*n*_ converges to zero faster than *d*_*n*_ to ensure the distinguishability of the null distribution. We further remark that the minimax rate for nonparametric estimation is *n*^−*m*/(2*m*+1)^ (Yang et al. (2017)) which is higher than the minimax distinguishable rate *n*^−2*m*/(4*m*+1)^. In the next section, we will introduce a Wald type test for the hypothesis testing (18) with the separation rate *d*_*n*_ achieves the lower bound *n*^−2*m*/(4*m*+1)^ indicating our proposed test is minimax optimal.

## 4. Wald Type Parallism Test

In this section, we propose a Wald type test statistics based on the penalized least squares estimate of *f*_11_, and derive the asymptotic distribution of the test statistics. We further prove an upper bound of the distinguishable rate of the Wald type test which matches the minimax lower bound established in Theorem 6.

### 4.1. Wald Type Test and Asymptotic Distribution

The nonparallel effect of the curves between the case group and the control group is measured by the magnitude of ${\left\|f_{11}\right\|}_{2}^{2}$. The nonparallel test in Equation (3) is equivalent to

$$H_{0}:f\in H_{\text{model}}^{\infty }⊖H_{11}^{\infty }\;vs\;H_{1}:f\in H_{\text{model}}^{\infty }$$

or equivalently, $H_{1}:f_{11}\in H_{11}^{\infty }$. First, notice that the null hypothesis in Equation (18) is a composite hypothesis as the null hypothesis defines a class of functions in $H_{\text{model}}^{\infty }⊖H_{11}^{\infty }$. Second, *H*_0_ defines an infinite dimensional parameter spaces as *n* → ∞, the assumptions of Neyman-Pearson Lemma cannot be satisfied. Thus the uniformly most powerful test may not exist in general. To overcome the difficulty, we propose a Wald-type test

(25) $$T_{n,\lambda }=\frac{1}{ns}{\left\|{\hat{f}}_{11}\right\|}_{2}^{2}$$

and show its minimax optimality.

Since *Y*_*ij*_ follows Equation (1) with *f* satisfying the SSANOVA decomposition in Equation (2), we can replace each element in vector **y** by $f_{00}\left(x_{i}^{\langle 1\rangle },x_{j}^{\langle 2\rangle }\right)+f_{10}\left(x_{i}^{\langle 1\rangle },x_{j}^{\langle 2\rangle }\right)+f_{01}\left(x_{i}^{\langle 1\rangle },x_{j}^{\langle 2\rangle }\right)+f_{11}\left(x_{i}^{\langle 1\rangle },x_{j}^{\langle 2\rangle }\right)+\epsilon_{ij}$. Then plug in the expression of ${\hat{f}}_{11}$ in Equation (17) to *T*_*n*,*λ*_, we have

$$T_{n,\lambda }=\frac{1}{ns}\|K_{11}M^{-1}\left(I_{n}-S{\left(S^{T}M^{-1}S\right)}^{-1}S^{T}M^{-1}\right){\left(f_{00}+f_{10}+f_{01}+f_{11}+\epsilon \right)\|}_{2}^{2},$$

where ***f***_00_, ***f***_10_, ***f***_01_ and ***f***_11_ are *ns* dimensional vectors with the *ij*th entry $f_{00}\left(x_{i}^{\langle 1\rangle },x_{j}^{\langle 2\rangle }\right)$, $f_{10}\left(x_{i}^{\langle 1\rangle },x_{j}^{\langle 2\rangle }\right)$, $f_{01}\left(x_{i}^{\langle 1\rangle },x_{j}^{\langle 2\rangle }\right)$ and $f_{11}\left(x_{i}^{\langle 1\rangle },x_{j}^{\langle 2\rangle }\right)$ respectively, and ***ϵ*** is the *ns* dimensional stochastic error that follows a normal distribution with mean 0 and variance *σ*^2^*I*_*ns*_. Because *f*_00_, *f*_10_ and *f*_01_ are in the space that is orthogonal to the space spanned by *K*_11_, and *f*_11_ = 0 under the null hypothesis, *T*_*n*,*λ*_ can be further simplified as

(26) $$T_{n,\lambda }=\frac{1}{ns}{\left\|K_{11}M^{-1}\left(I_{ns}-S{\left(S^{T}M^{-1}S\right)}^{-1}S^{T}M^{-1}\right)\epsilon \right\|}_{2}^{2}.$$

A detailed discussion of this simplification will be provided in Lemma 12 in Appendix.

Next, we develop the null limiting distribution of *T*_*n*,*λ*_ as *n* goes to infinity. In the derivation, we only require the number of subjects *s* to be finite. This requirement is desired in real applications since the number of subjects in an experiment is usually limited. For example, due to the high sequencing cost, there are usually only tens of sample sequenced in the DNA methylation studies.

We consider the following two designs.

**Quasi-Uniform Design :** $x_{1}^{\langle 1\rangle },x_{2}^{\langle 1\rangle },\ldots ,x_{n}^{\langle 1\rangle }\overset{\text{iid}}{\sim }\omega \left(x^{\langle 1\rangle }\right)$ where *ω* is the marginal density of *x*^〈1〉^. For any *x*^〈1〉^ ∈ [0, 1], there exist two constants *c*_1_, *c*_2_ > 0 such that *c*_1_ ≤ *ω*(*x*^〈1〉^) ≤ *c*_2_ (Eggermont and LaRiccia, 2001).

**Uniform Design:** $x_{1}^{\langle 1\rangle },x_{2}^{\langle 1\rangle },\ldots ,x_{n}^{\langle 1\rangle }$ are evenly spaced on [0, 1].

The above two designs are commonly used in scientific investigations. For example, in fMRI experiments, the sampling points on the time domain are usually measured with equal-time intervals. Thus, they are assumed to follow uniform design. On the other hand, the DNA methylation sites are randomly scattered on DNA sequence. Therefore, they are assumed to follow a quasi-uniform design.

**Theorem 7** *For both the uniform design and the quasi-uniform design, if the smoothing parameter* $\lambda =O\left(n^{c-1}\right)$ *for any fixed c* ∈ (0, 1), *we have*

$$\frac{T_{n,\lambda }-\mu_{n,\lambda }}{\sigma_{n,\lambda }}\overset{d}{\to }N(0,1)\;\text{ as }n\to \infty ,$$

*where μ*_*n*,*λ*_ = *σ*^2^ Tr(Δ)/(*ns*) *and* $\sigma_{n,\lambda }^{2}=2\sigma^{4}\text{Tr}\left(\Delta^{2}\right)/{\left(ns\right)}^{2}$ *with* $\Delta =M^{-1}K_{11}^{2}M^{-1}$.

In practice, we estimate the variance *σ*^2^ via ${\hat{\sigma }}^{2}$ defined as

$${\hat{\sigma }}^{2}=\frac{y^{⊤}{\left(I-A(\lambda )\right)}^{2}y}{\text{Tr}(I-A(\lambda ))},$$

where *A*(*λ*) = *K*(*nsK*^2^ + *λQ*)^−1^**y**, and (*I* − *A*(*λ*))**y** is the residual $y-\hat{f}$ based on the objective function in equation (15). The consistency of the variance estimate ${\hat{\sigma }}^{2}$ is established in Theorem 3.4 in Gu (2013).

The proof of Theorem 7 is provided in Appendix and sketched below. Notice that *T*_*n*,*λ*_ = *T*_1_ + *T*_2_ − 2*T*_3_, where

(27) $$T_{1}=\frac{1}{ns}\epsilon^{T}M^{-1}K_{11}^{2}M^{-1}\epsilon ,\;T_{2}=\frac{1}{ns}{\left\|K_{11}M^{-1}S{\left(S^{T}M^{-1}S\right)}^{-1}S^{T}M^{-1}\epsilon \right\|}_{2}^{2},\;T_{3}=\frac{1}{ns}\epsilon^{T}M^{-1}S{\left(S^{T}M^{-1}S\right)}^{-1}S^{T}M^{-1}K_{11}^{2}M^{-1}\epsilon .$$

We show that *T*_2_ and *T*_3_ are higher order small perturbation terms compared to *T*_1_. Thus, the null distribution of *T*_*n*,*λ*_ and the distribution of *T*_1_ are asymptotically equivalent. We only need to focus on the distribution of the quadratic form $T_{1}=\frac{1}{ns}\epsilon^{T}\Delta \epsilon$ with ***ϵ*** having a mean zero normal distribution. To prove the normality of *T*_1_, we show that the log-characteristic function of the standardized *T*_1_ is asymptotically −*σ*^2^*t*^2^/2, provided that Tr(Δ^2^) diverges as *λ* → 0. Lemma 15 shows that $\text{Tr}\left(\Delta^{2}\right)≽{\hat{\tau }}_{\lambda }$, where ${\hat{\tau }}_{\lambda }=\text{max}\left\{i|{\hat{\mu }}_{i}\geq \lambda \right\}$ as the effective dimension (Bartlett et al., 2005; Liu et al., 2019) with ${\hat{\mu }}_{1}\geq \cdots \geq {\hat{\mu }}_{n}$ the empirical eigenvalues of kernel matrix $K_{1}^{\langle 1\rangle }$ which is the kernel matrix of $H_{1}^{\langle 1\rangle }$ with (*i*, *i*′)th entry as $\frac{1}{n}K_{1}^{\langle 1\rangle }\left(x_{i}^{\langle 1\rangle },x_{i^{\prime }}^{\langle 1\rangle }\right)$. We further show in Lemma 13 and 14 that ${\hat{\tau }}_{\lambda }$ is of the same order as its population counterpart *τ*_*λ*_ defined as *τ*_*λ*_ = max{*i* | *μ*_*i*_ ≥ *λ*}, under both the quasi-uniform design and the uniform design, where *μ*_1_ ≥ ⋯ ≥ 0 are a sequence of ordered eigenvalues satisfying $K_{1}^{\langle 1\rangle }\left(x,x^{\prime }\right)=\sum_{i=1}^{\infty }\mu_{i}\phi_{i}(x)\phi_{i}\left(x^{\prime }\right)$. Since *μ*_*i*_ has a polynomial decay rate *i*^−2*m*^ (Gu, 2013), we have $\text{Tr}\left(\Delta^{2}\right)≽{\hat{\tau }}_{\lambda }≍\tau_{\lambda }≍\lambda^{-1/(2m)}$ diverges as *λ* → 0. Consequently, the testing consistency in Theorem 7 holds.

Theorem 7 characterizes the distribution of the test statistic *T*_*n*,*λ*_ for $f\in H_{\text{model}}^{\infty }⊖H_{11}^{\infty }$. The distribution turns out to be fairly simple and easy to calculate as the test statistic does not depend on any unknown nuisance functions such as *f*_00_, *f*_10_ and *f*_01_. Critical value can be easily found based on the known null distribution $N\left(\mu_{n,\lambda },\sigma_{n,\lambda }^{2}\right)$. Consequently, one can make a statistical decision by comparing *T*_*n*,*λ*_ with the critical value. This nuisance-parameter free property is referred to as the “Wilks phenomenon” in statistics literature (Fan et al., 2001; Fan and Zhang, 2004).

### 4.2. Upper Bound of the Distingushiable Rate

Given type I error *α*, we show that our Wald-type testing rule $\phi_{n,\lambda }=1_{\left(\left|T_{n,\lambda }-\mu_{n,\lambda }\right|\geq z_{\alpha /2}\sigma_{n,\lambda }\right)}$ achieves the local minimax distinguishable rate. Without loss of generality, we assume $\|f{\|}_{H}\leq 1$.

**Theorem 8** *Let the minimum distinguishable rate of the test ϕ*_n,λ_ *be d*_*n*_(*ϕ*_n,λ_). *Suppose* $\lambda =O\left(n^{c-1}\right)$ *for any fixed c* ∈ (0, 1)*. Then for any δ* > 0*, there exist positive constants C*_*δ*_ *and N*_*δ*_ *such that, when n* ≥ *N*_*δ*_*, the tolerable pseudo.risk*(*ϕ*_n,λ_, *d*_*n*_) = *α* + *δ, with* $d_{n}\left(\phi_{n,\lambda }\right)≔C_{\delta }\sqrt{\lambda +\sigma_{n,\lambda }}$.

Theorem 8 shows that for a controlled type I error, *T*_*n*,*λ*_ can achieve arbitrary small type II error provided that the local alternative is separated from the null by at least an amount of *d*_*n*_(*ϕ*_*n*,*λ*_). The proof of Theorem 8 is collected in Appendix.

Note that $d_{n}^{2}\left(\phi_{n,\lambda }\right)$ consists of two components: *σ*_*n*,*λ*_ representing the standard variation of the test statistic *T*_*n*,*λ*_, and *λ* representing the squared bias of ${\hat{f}}_{1,2}$ (see the proof of Lemma S.1 in the Supplementary). Through approximating *σ*_*n*,*λ*_ by the Rademacher complexity (Bartlett et al., 2005; Liu et al., 2019), we show that $\sigma_{n,\lambda }≍\sqrt{\tau_{\lambda }}/n$, which is a decreasing function of *λ*. Hence, the minimum distinguishable rate for *ϕ*_*n*,*λ*_ is achieved by the trade-off between the bias of ${\hat{f}}_{1,2}$ and the standard derivation of *T*_*n*,*λ*_, i.e., choosing appropriate *λ* such that *λ* ≍ *σ*_*n*,*λ*_. Next, we prove that our proposed Wald-type test is minimax under two special design conditions: the quasi-uniform design and the uniform design in the next two corollaries.

**Corollary 9 *[Quasi-Uniform Design]*** *Let λ* ≍ *n*^−4m/(4m+1)^ *and suppose x*^〈1〉^ *follows a quasi-uniform design. We have*

$$P\left(d_{n}\left(\phi_{n,\lambda }\right)≍n^{-2m/(4m+1)}\right)\geq 1-4\text{ exp}\left(-n^{1/(2m+1)}\right).$$

**Corollary 10 *[Uniform Design]*** *Let λ* ≍ *n*^−4m/(4m+1)^*, and suppose x*^〈1〉^ *follows a uniform design, we have*

$$d_{n}\left(\phi_{n,\lambda }\right)≍n^{-2m/(4m+1)}\;a\text{.}s\text{.}$$

Corollaries 9 and 10 suggest that if *λ* ≍ *n*^−4*m*/(4*m*+1)^, our Wald-type test *ϕ*_*n*,*λ*_ can achieve the minimax distinguishable rate $d_{n}^{†}≍n^{-2m/(4m+1)}$. Thus, we demonstrate that our proposed Wald type test is minimax optimal. We remark that Corollary 9 still holds when extending $H^{\langle 1\rangle }$ as a standard Sobolev space.

### 4.3. The Choice of Regularization Parameter

Different from the classical “bias-variance” tradeoff in optimal nonparametric estimation, Theorem 8 states that the optimal nonparametric testing for Equation (3) can be achieved by another type of tradeoff between the squared bias of the estimator and the standard deviation of the test statistic. Such intrinsic difference further leads to different orders of optimal regularization parameters: as shown in Corollary 9, 10, the optimal *λ* is chosen as the order of $n^{-\frac{4m}{4m+1}}$; while as the order of $n^{-\frac{2m}{2m+1}}$ for optimal estimation (Gu, 2013).

In practice, cross validation method is often used as a tuning procedure for nonparametric estimation based on penalized loss functions (Golub et al., 1979). Raskutti et al. (2014) proposed another data-dependent algorithmic regularization technique, that is, choosing an early stopping rule for an iterative algorithm to avoid over-fitting in nonparametric estimation. Both of the above approaches are optimal for estimation but suboptimal for testing. There has few theoretically justified tuning procedure for obtaining optimal testing in nonparametric inference. One related work we are aware currently is Liu and Cheng (2018), under which they developed a data-dependent early stopping regularization rule from an algorithmic perspective for testing *f* = 0 in nonparametric regression model *Y* = *f*(*X*) + *ϵ*. The total step size determined via the early stopping rule in gradient descent algorithm plays the same role with 1/*λ* in the penalized regularization, to avoid over-fitting. However, a data-adaptive choice of the regularization parameter *λ* is still lacking for nonparametric inference in Equation (3) under the penalization regularization.

We propose a data-adaptive method to choose *λ* with testing optimality guarantee based on Theorem 8. In practice, we can choose the optimal smoothing parameter λ* satisfying

(28) $$\lambda *=\text{min }\left\{\lambda |\lambda <\sigma_{n,\lambda }\right\},$$

where *σ*_*n*,*λ*_ can be explicitly calculated based on the observed data by the expression defined in Theorem 7, i.e., $\sigma_{n,\lambda }^{2}=2\sigma^{4}\text{Tr}\left(\Delta^{2}\right)/{\left(ns\right)}^{2}$, with $\Delta =M^{-1}K_{11}^{2}M^{-1}$.

The above criterion in Equation (28) in choosing *λ* is a data-dependent rule that produces a minimax-optimal nonparametric testing method. Based on the Rademacher complexity, $\sigma_{n,\lambda }≍\frac{\sigma^{2}}{ns}\sqrt{\sum_{i=1}^{n}\text{min}\left\{1,{\hat{\mu }}_{i}/\lambda \right\}}$. That is, the rule in Equation (28) depends on the eigenvalues of the kernel matrix, especially the first few leading eigenvalues. There are many efficient methods to compute the top eigenvalues fast (Drineas and Mahoney, 2005; Ma and Belkin, 2017). As a future work, we can also introduce the randomly projected kernel methods to accelerate the computing time.

## 5. Simulation Study

To assess the performance of our proposed test, we carried out extensive analyses on simulated data sets. We compared our approach with F-test (SSF) (Ma et al., 2009), parallelism trend test (PTT) (Degras et al., 2011) and a random permutation test with 500 permutations. In the three methods, permutation test can be used as a benchmark because it can closely approximate null distribution when the number of permutations is adequate. However, the permutation test is computationally intensive, especially for calculating the Kullback-Leibler distance under the null and alternative hypothesis for SSANOVA model (Gu, 2004).

### 5.1. Empirical Power Analysis

**Figure 3:**
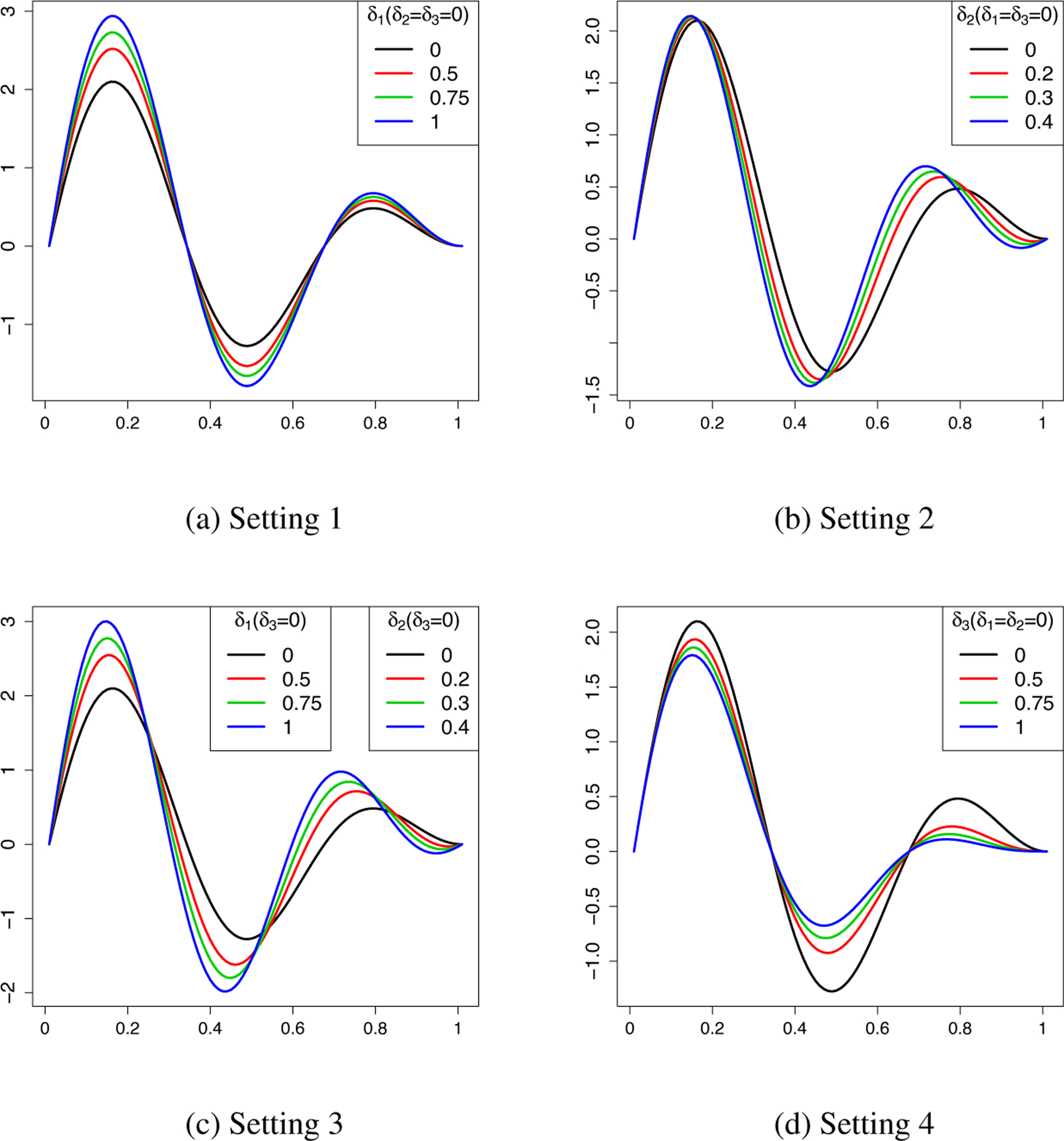
Plotted here are functions of the control group (solid line) and case group (dashed, dotted and dot-dash lines) with four types of nonparallel patterns: magnitude differences only (Setting 1), frequency differences only (Setting 2), both magnitude and frequency differences (Setting 3), and magnitude dynamic differences (Setting 4).

We illustrate the empirical power performance of our proposed test through four well-designed examples. In all four examples, we generated 100 to 1000 observations with an increment of 100 observations in each simulation for both case and control groups in Equation (1), where $x_{i}^{\langle 1\rangle }\overset{\text{iid}}{\sim }U\left(0,1\right)$ and $\epsilon_{ij}\overset{\text{iid}}{\sim }N(0,1)$. Each example was repeated 500 times for power and other comparisons. To make the simulation more close to the reality, we considered two types of nonparallel patterns between *f*(*x*^〈1〉^, 1) and *f*(*x*^〈1〉^, 0): magnitude and frequency. These two kinds of nonparallel patterns are often observed in real applications. For example, the hypermethylated DNA regions, i.e., regions with low methylation levels, are related to transcriptional silencing which plays an important role in cancer development; the frequency differences are often related to different brain functions between the neurodisease and control groups in fMRI studies. In the first four examples, we consider the following function in Equation (1),

(29) $$f\left(x^{\langle 1\rangle },x^{\langle 2\rangle }\right)=\{\begin{array}{ll} 2.5\text{ sin}\left(3\pi x^{\langle 1\rangle }\right)\left(1-x^{\langle 1\rangle }\right) & \text{ if }x^{\langle 2\rangle }=0,\text{ i.e., control}\\ \left(2.5+\delta_{1}\right)\text{ sin}\left(\left(3+\delta_{2}\right)\pi x^{\langle 1\rangle }\right){\left(1-x^{\langle 1\rangle }\right)}^{\left(1+\delta_{3}\right)} & \text{ if }x^{\langle 2\rangle }=1,\text{ i.e., case} \end{array}$$

where *δ*_1_, *δ*_2_ and *δ*_3_ control the magnitude of nonparallelism between the null hypothesis and the alternative hypothesis in Equation (18). In general, varying *δ*_1_, *δ*_2_ and *δ*_3_ give rise to different distinguishable rates *d*_*n*_s. The larger the *δ*_1_, *δ*_2_ and *δ*_3_ are, the larger the *d*_*n*_ is. To illustrate how the testing power is affected by different *δ*’s, as shown in Figure 3, we considered the following four settings. **Setting 1:** Case and control have constant magnitude differences (*δ*_1_ = 0.50, 0.75, 1.00 and *δ*_2_, *δ*_3_ = 0.00); **Setting 2:** Case and control have frequency differences (*δ*_2_ = 0.20, 0.30, 0.40 and *δ*_1_, *δ*_3_ = 0.00); **Setting 3:** Both magnitude and frequency are different (*δ*_1_, *δ*_2_ = (0.50, 0.20), (0.75, 0.30), (1.00, 0.40) and *δ*_3_ = 0.00); **Setting 4:** Case and control have non-constant magnitude differences (*δ*_1_, *δ*_2_ = 0.00 and *δ*_3_ = 0.50, 0.75, 1.00). The corresponding functions *f*(*x*^〈1〉^, 0) and *f*(*x*^〈1〉^, 1) are shown in Figure 3.

The empirical powers of our proposed Wald-type test, permutation test, SSF test and PTT test are summarized in Tables 1–2 for Settings 1–2. For Setting 1, as shown in Table 1, the empirical power of our test increases rapidly as sample size increases, and approaches to 1 even for the smallest magnitude (*δ*_1_ = 0.50). The empirical powers of the proposed test are comparable with that of the permutation test. In contrast, the empirical powers of SSF and PTT increase slower than our proposed test. For the weak signal scenario, i.e., *δ*_1_ = 0.50, the proposed test has significantly gain of power under different sample sizes. For the strong signal scenario, i.e, *δ* = 1.00, our proposed test is significantly more powerful than SSF and PTT when sample size is less than 500. For Setting 2, as shown in Table 2, the empirical power of our proposed test converges to 1 as the sample size increases for all three cases with *δ*_2_ = 0.20, 0.30 and 0.40. In contrast, the empirical power of SSF and PTT converges to 1 slower than the proposed test.

For Settings 3 and 4, we only included the empirical results for our proposed test and SSF test due to the extremely high computational cost of the permutation test. As shown in Table 6, it takes more than 150 hours to complete the permutation test for one setting. For Setting 3, we simulated the signal with differences in both scale and frequency across case and control groups. The empirical powers of the simulation with different distinguishable parameters are listed in Table 3. The empirical powers of our proposed test and SSF increase for all the three cases with *δ*_1_, *δ*_2_ = 0.20, 0.30, 0.40. The empirical power of PTT also increases, but with a much slower pattern. When the sample size is small and signal strength is weak, our proposed test has significant gain of power compared to the SSF and PTT test. For Setting 4, there is a nonlinear magnitude difference along the *x*^〈1〉^ between the two groups. As shown in Table 4, the empirical power of SSF test converges to one slower than the proposed test and is lower than 0.65 for the least distinguishable case.

### 5.2. Empirical Size Analysis

To examine the approximation of significance levels, we generated data from a new setting **Setting 5**. We kept the function form of control group the same as Equation (5.4) and only added a parallel shift over the control function as the function of the case group, i.e., the model does not include the nonparallel patterns. In particular,

$$f\left(x^{\langle 1\rangle },x^{\langle 2\rangle }\right)=2.5\text{ sin}\left(3\pi x^{\langle 1\rangle }\right)\left(1-x^{\langle 1\rangle }\right)+\delta_{4}I_{\left\{x^{\langle 2\rangle }=1\right\}},$$

where *δ*_4_ was set to be 0, 0.5 and 1 to characterize different level parallel difference in the two groups. We generated data from Equation (1) with function *f* specified in Setting 5. The rest of parameters were set the same as before.

Table 5 lists the empirical sizes of our proposed test, permutation test, SSF test, and PTT under Setting 5. We varied *δ*_4_ from 0.00 to 1.00 to model different magnitudes of the main effect. The empirical size of our proposed test approaches to 0.05 as the sample size increases for different values of *δ*_4_. The empirical size of SSF test is fluctuating from 0.03 to 0.1. The inaccurate size of the SSF test may be attributed to the fact that the degrees of freedom of the SSF test is very roughly approximated by the rounding value of the trace of the smoothing matrix. The empirical size of PTT test is fluctuating from 0.02 to 0.12.

### 5.3. Computation Time

As shown in Tables 1 and 2, our purposed test achieves the power similar to the permutation test. Next, we compared the computation time of our proposed test and permutation test for 500 replicated samples. We conducted the comparison on a computer workstation with core Intel i7 8700k CPU and 32 Gb RAM. In Table 6, we reported the computational time in Setting 1 with *δ*_1_ = 0.5 and sample size ranging from 100 to 1000. As shown in Table 6, our proposed test is consistently faster than the permutation test. Our proposed test is nearly 263×faster than the permutation test when the sample size is 1000. Note that the computational time is more than 42 hours when the sample size is 1000 for running 500 test. In practice, the huge computational cost limits the application of the permutation test in many large scale studies involving large sample size and multiple tests.

### 5.4. Simulation Studies with Correlated Noise

We established **Setting 6** to evaluate the performance of the proposed test when the noises are correlated. In this example, we generated 100 to 1000 observations with an increment of 100 observations in each simulation for both case and control groups in Equation (1). We considered $x_{i}^{\langle 1\rangle }$, *i* = 1, …, *n* are evenly distributed in [0, 1]. We generated two correlated noise vector (*ϵ*_11_, …, *ϵ*_*n*1_) and (*ϵ*_12_, …, *ϵ*_*n*2_) i.i.d. from *N*(0, Σ) where Σ is autoregressive, i.e., each of its element *σ*_*ii*′_ = *ρ*^|*i*−*i*′|^ with *ρ* = 0.5. We generated the signal $Y_{ij}=f\left(x_{i}^{\langle 1\rangle },x_{j}^{\langle 2\rangle }\right)+\epsilon_{ij}$ where *f* is defined in Equation (5.4) with *δ*_1_ = 0.00, 0.50, 0.75, 1.00 and *δ*_2_, *δ*_3_ = 0.00, that is,

$$f\left(x^{\langle 1\rangle },x^{\langle 2\rangle }\right)=\{\begin{array}{ll} 2.5\text{ sin}\left(3\pi x^{\langle 1\rangle }\right)\left(1-x^{\langle 1\rangle }\right) & \text{ if }x^{\langle 2\rangle }=0,\\ \left(2.5+\delta_{1}\right)\text{ sin}\left(3\pi x^{\langle 1\rangle }\right)\left(1-x^{\langle 1\rangle }\right) & \text{ if }x^{\langle 2\rangle }=1. \end{array}$$

We set the significance level as 0.05 and repeated 500 times for evaluating the empirical size and power.

As shown in Table 7, when *δ*_1_ = 0.00, the size of our proposed method concentrates around 0.05 − 0.07, while the sizes of SSF and PTT are fluctuating from 0.02 to 0.16. When *δ*_1_ > 0.00, compared with SSF and PTT, the power of our proposed method has the highest performance, and approaches to 1 as *δ*_1_ increases.

### 5.5. Simulation Studies with Non-smooth Cases

**Figure 4:**
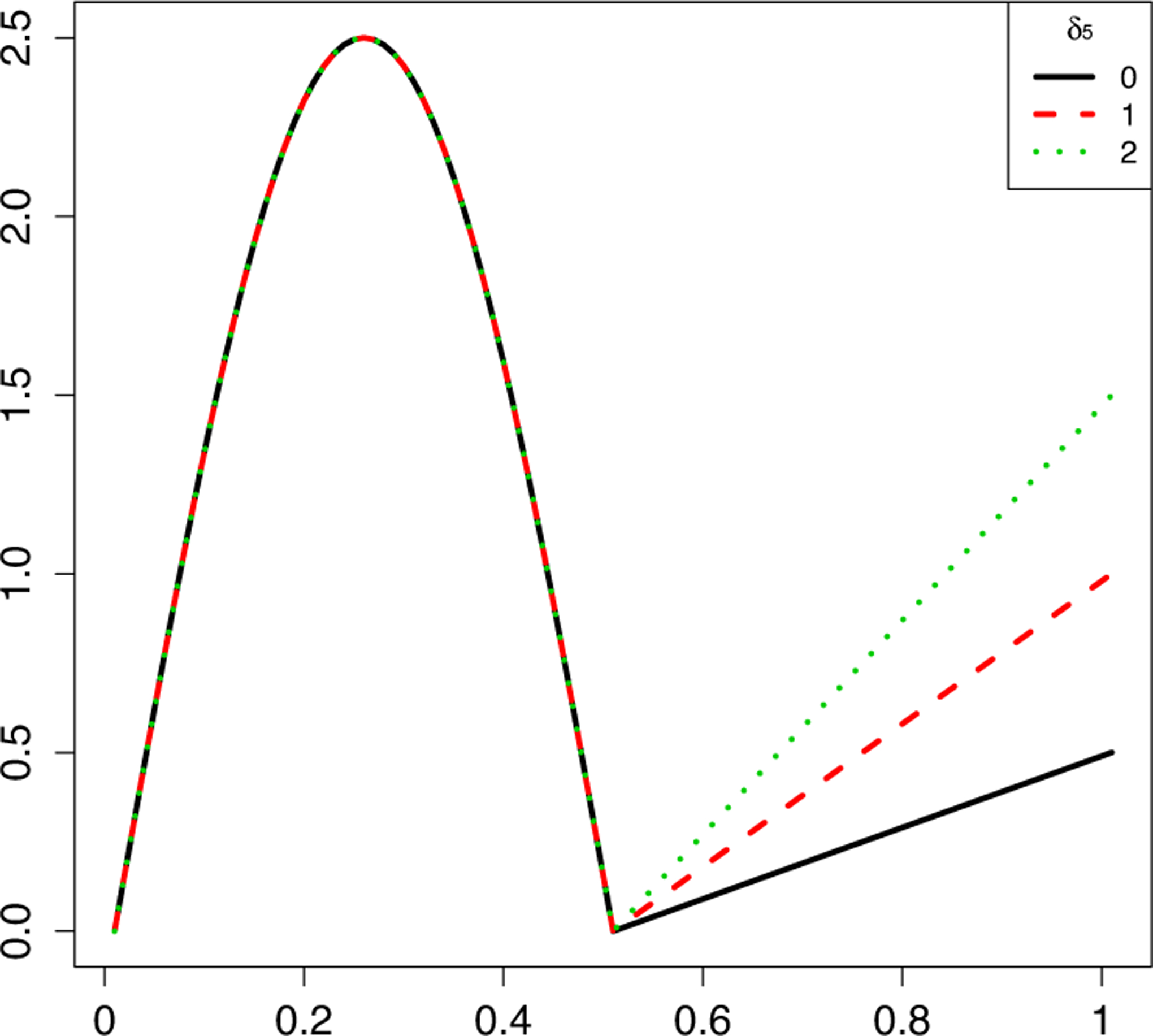
Solid line with *δ*_5_ = 0: function of the control group; dashed and dotted lines with *δ*_5_ = 1, 2: the case group for Setting 7.

We evaluate the robustness of the proposed method when the smoothness assumption is invalid. We established **Setting 7** to test the performance of the proposed test for the cases with non-smooth trends. In this setting, we generated 100 to 1000 observations with an increment of 100 observations in each simulation for both case and control groups in model (1). We considered $x_{i}^{\langle 1\rangle }$, *i* = 1, …, *n*, are evenly distributed in [0, 1] and $\epsilon_{ij}\overset{\text{iid}}{\sim }N(0,1)$. We generated the signal $Y_{ij}=f\left(x_{i}^{\langle 1\rangle },x_{j}^{\langle 2\rangle }\right)+\epsilon_{ij}$ with *f* defined as

$$f\left(x^{\langle 1\rangle },x^{\langle 2\rangle }\right)=2.5\text{ sin}\left(2\pi x^{\langle 1\rangle }\right)I_{\left\{x^{1}\in (0,0.5)\right\}}+\left(1+\delta_{5}I_{\left\{x^{\langle 2\rangle }=1\right\}}\right)(x-1)I_{\left\{x^{\langle 1\rangle }\in [0.5,0)\right\}}$$

which is shown in Figure 4. This curve is non-differentiable at *x*^〈1〉^ = 0.5 which is a change point from nonlinear to linear trend. We set the significance level as 0.05 and repeated 500 times to evaluate the empirical size and power.

As shown in Table 8, when *δ*_5_ = 0.00, the empirical size of our proposed method concentrates around 0.05. The empirical size of our proposed method is slightly inflated compared with SSF and PTT. When *δ*_5_ = 1, 2, compared with SSF and PTT, the power of our proposed method has the highest performance, and approaches to 1 as *n* increases.

## 6. Real Data Examples

We apply the technique to analyze two real data sets: DNA methylation in chronic lymphocytic leukemia and neuroimaging of Alzheimer’s Disease using fMRI.

### 6.1. DNA Methylation in Chronic Lymphocytic Leukemia

Recently, Filarsky et al. (2016) reported a DNA methylation study for chronic lymphocytic leukemia (CLL) patients. In the study, the DNA samples were extracted from CD19+ cells from 12 CLL patients and B cells from 6 normal subjects. The DNA methylation is profiled by the whole-genome tiling array technique. The goal is to identify differentially methylated regions (DMRs), i.e., the genome regions that have significantly different methylation levels, between CLL patients and normal subjects.

To achieve this goal, we compiled the DNA methylation intensities within the −3.8 to +1.8 kb of transcription start sites (TSS) for each gene. We used the M-value suggested by Irizarry et al. (2008) as methylation level at each site and as our response variable. In particular, the data consists of $\left(Y_{ij},x_{i}^{\langle 1\rangle },x_{j}^{\langle 2\rangle }\right)$, where *Y*_*ij*_ is the methylation level at the *i*th genome location $x_{i}^{\langle 1\rangle }$ of the *j*th subject in group $x_{j}^{\langle 2\rangle }$, which equals to 1 if the *j*th subject is in the case group and equals to 0 if the *j*th subject is in the control group. We fit the model in Equation (1) with SSANOVA decomposition in Equation (2) to the data.

We applied the proposed hypothesis testing on 10383 regions. Through controlling FDR < 0.01 using Benjamini-Hochberg procedure (Benjamini and Hochberg, 1995), we selected 613 DMRs. We conducted gene ontology analysis on the 613 genes corresponding 613 identified DMRs using the GSEA (Subramanian et al., 2005). Among these genes, 79 genes participate the lipid metabolic process, which plays an important role in the development of CLL (Pallasch et al., 2008). This biological process contributes to apoptosis resistance in CLL cells. Furthermore, 78 and 61 genes participate the immune related biological processes: “Immune system process” and “Regulation of immune system process” respectively. The observation indicates that the aberrant DNA methylation has the potential impact on the immune system.

Our Wald-type test, even after FDR control, yields p-values that are as small as 10^−9^. Consequently, it is very difficult to compare our test with the permutation test with only hundreds or thousands of permutations. Thus, we only compared our proposed test with permutation test (based on 500 permutation) for regions with p-values larger than 0.05 the averaged difference between our test and permutation test is 0.012.

**Figure 5:**
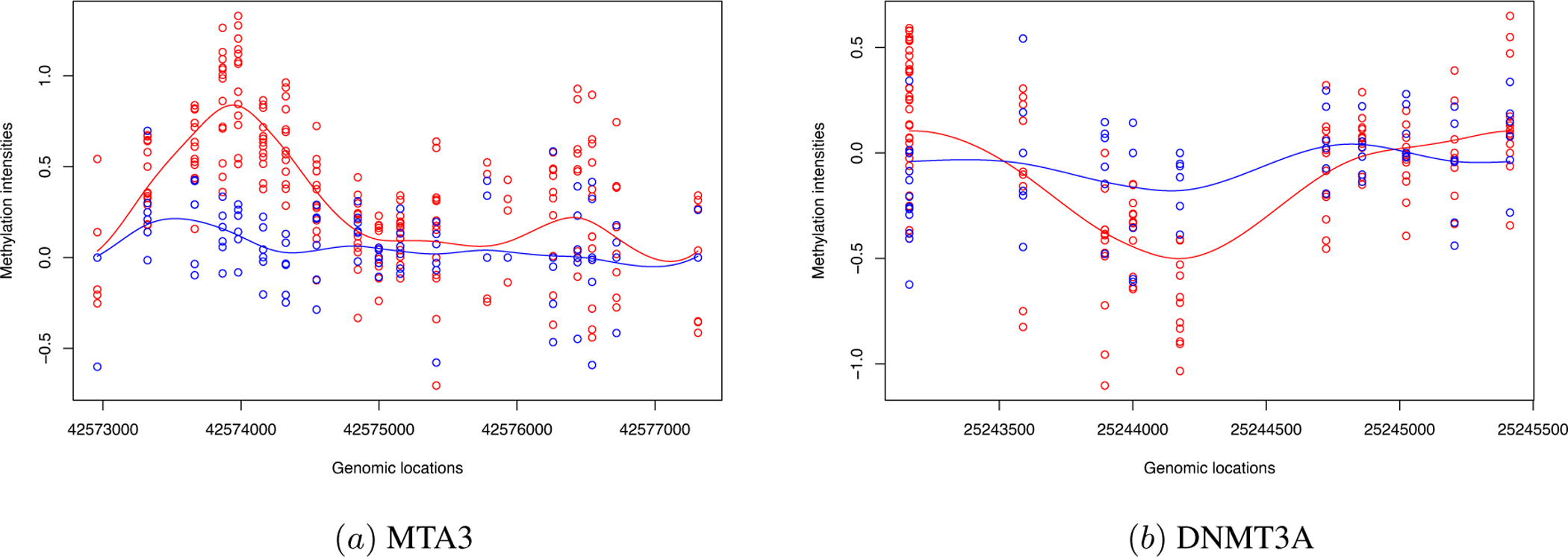
The promoter regions of two genes, (a) MTA3 and (b) DNMT3A. The horizontal axis is the genomic location and the y axis is the M-value representing the methylation levels. The red and blue lines are the fitted curves for the case and control groups respectively.

We highlighted two DMRs with significant nonparallel patterns in Figure 5. The focal hypermethylation at genome locations 42574000 and 42576500 are observed on the promoter region of gene MTA3. It was reported in (Bilban et al., 2006) that MTA3 signaling pathway is a potential bio-marker for CLL and shows significantly altered gene expression. Our test also identified that the methylation levels between CLL patients and normal subjects, of MTA3 gene have significant difference, which has potential prognostic value. In the promoter region of DNMT3, we observed significant hypomethylation at genome location 25244500. DNMT3 is a family of DNA methyltransferases that could methylate hemimethylated and unmethylated CpG sites at the same rate (Okano et al., 1998). Since the global hypomethylation is observed, the aberrant methylation levels of this DNA methylatransferase may have influence on this global trend.

### 6.2. Neuroimaging of Alzheimer’s Disease using fMRI

Alzheimer’s disease (AD) is one of the most commonly known neurology disease characterized with neurodegeneration and cognitive decline (Rombouts et al., 2005; Wang et al., 2006). Despite the prevalence of AD, there are no cure or preventive methods available due to the lack of a complete understanding of the mechanisms that contribute to AD pathophysiology. Discovering aberrant neural network of AD will fundamentally advance the scientific understanding of this disease.

In this study, we analyzed the data that was collected by Alzheimer’s Disease Neuroimaging Initiative (ADNI)^2^, in which the resting-state fMRI signals of 60 normal/early-mild-cognitive-impairment subjects (control group) and 50 AD/late-mild-cognitive-impairment subjects (AD group) were collected from 256×256×170 voxels for 140 consecutive time points with equal time intervals of 30ms. The fMRI signals for each subject were preprocessed using fMRI Expert Analysis Tool (FEAT) (Smith et al., 2004) for skull-stripping, motion correction, slice timing correction, temporal filtering, spatial smoothing and registration to standard space (MNI152 T1 2mm model) so that signals from all subjects can be considered as from the same engineered brain template. Sixty-nine brain-region-of-interests (ROI) that are defined by Harvard-Oxford-Atlas (http://fsl.fmrib.ox.ac.uk/fsl/fslwiki/Atlases) was extracted by automatic regional labeling approach using the refined fMRI data. For each ROI, we consider model (1) with SSANOVA decomposition in Equation (2), where *Y*_*ij*_ records the average blood-oxygen-level (Huettel et al., 2004) of the brain region for subject *j* measured at the $x_{i}^{\langle 1\rangle }$ time point. As the blood-oxygen-level can accurately quantify the corresponding brain activity, we can detect abnormal AD related brain activity. Testing problem in Equation (18) is equivalent to testing whether the brain activities of a given ROI have different temporal patterns in case and control groups.

**Figure 6:**
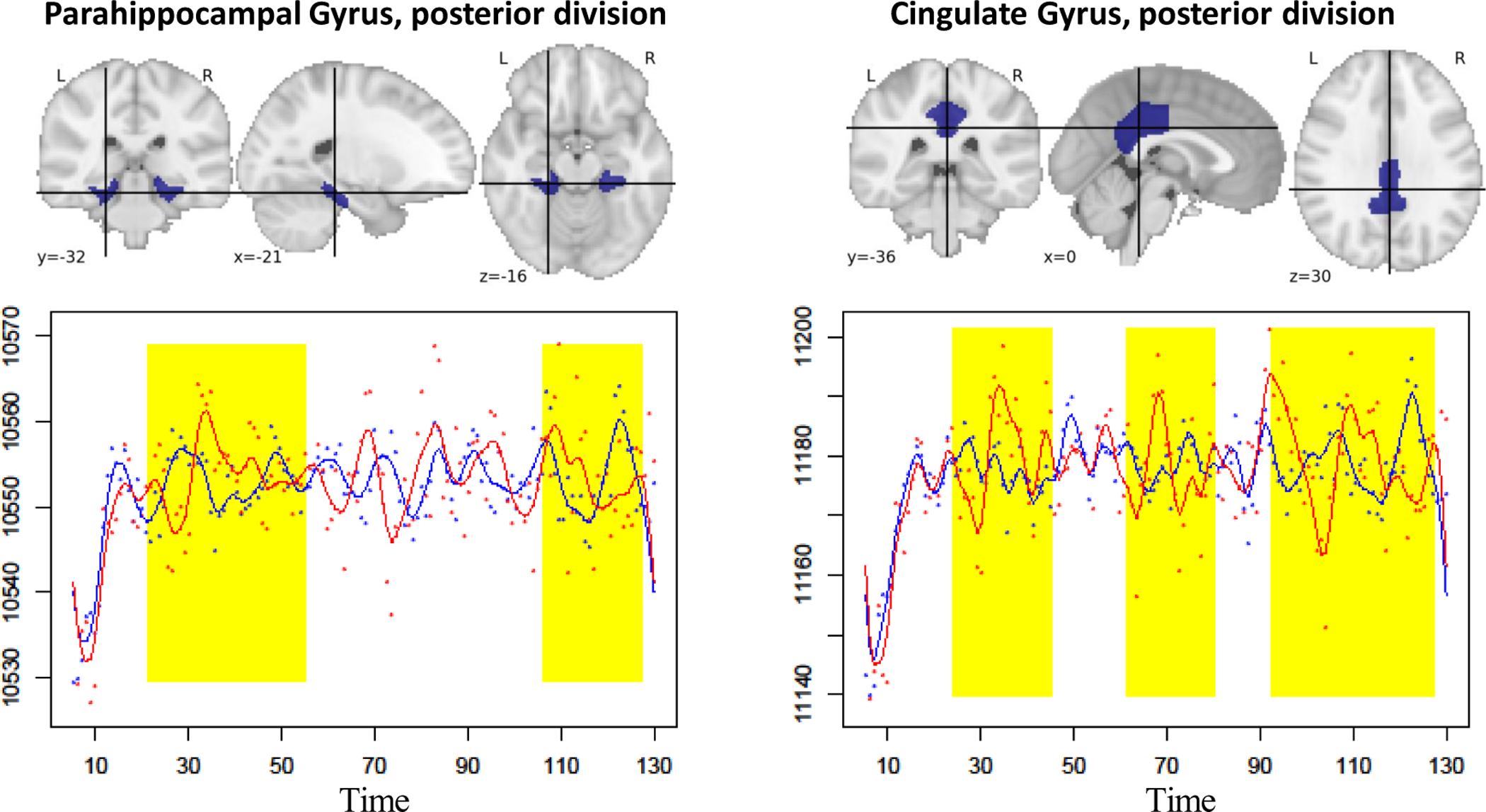
Plotted here are blood-oxygen-levels of *parahippocampal gyrus* (left) and *cingulate gyrus* (right) for control group (blue) and AD group (red) observed at 140 time points. Physical locations of either ROIs on frontal, axial and lateral sides are illustrated on the top of each panel.

Seven cortical regions *parahippocampal gyrus*, *cingulate gyrus*, *inferior temporal gyrus*, *post-central gyrus*, *juxtapositional lobule cortex*, *precuneous cortex*, *central opercular cortex* and one sub-cortical region *right thalamus* with significantly different temporal patterns were identified using our test with the false discovery rate controlled at 5% using Benjamini-Hochberg procedure (Benjamini and Hochberg, 1995). Among the eight ROIs, *parahippocampal gyrus* and *cingulate gyrus* have been shown clinically to be risk factors for AD. As demonstrated in Echávarri et al. (2011) and Kesslak et al. (1991), *parahippocampal gyrus* of AD patients have significant atrophy. Meanwhile, *cingulate gyrus* was also found to be AD related (Scheff et al., 2015) due to its extensive connectivity with multiple different cortical areas, especially areas involved with learning and memory. In Figure 6, we plotted frontal, axial, and lateral views and corresponding temporal patterns of *parahippocampal gyrus* and *cingulate gyrus*. The temporal regions with significant difference between AD/late-mildcognitive-impairment subjects (red line) and normal/early-mild-cognitive-impairment subjects (blue line) are highlighted. As clearly demonstrated in lower left panel of Figure 6, the first highlighted area of *parahippocampal gyrus* has a significant reversed pattern between case group and control group. The second highlighted area shows the reduced levels for the AD group. For *cingulate gyrus*, the highlighted regions in the right panel of Figure 6 show clearly larger magnitude for the AD groups. This difference was also observed via fMRI in a visual encoding memory task (Rami et al., 2012). Both of the two experiments suggest that the difference may change the memory function.

## 7. Discussion

The hypothesis testing in SSANOVA is a very challenge problem. In this paper, we develop a Wald-type test for testing the significance of the nonparallelism in a two-way SSANOVA model. The optimality of the proposed test is justified by the minimax distinguishable rate. The extensive empirical studies suggest that the proposed test has a superior performance over existing methods. Although we only discuss the test of the significance of the nonparallelism in a two-way SSANOVA model, the test on a higher order SSANOVA model can be developed parallel to our framework.

## Supplementary Material

1

## Figures and Tables

**Table 1:** Table lists the empirical power of our proposed test and permutation test for Setting 1 with *δ*_1_ = 0.50, 0.75, 1.00, *δ*_2_ = *δ*_3_ = 0.00 and sample size ranging from 100 to 1000.

		Sample Size									
		100	200	300	400	500	600	700	800	900	1000
*δ*_1_ = 0.50	Proposed	0.17	0.33	0.49	0.59	0.69	0.75	0.86	0.91	0.92	0.96
	Permutation	0.19	0.38	0.53	0.60	0.62	0.76	0.80	0.88	0.94	0.97
	SSF	0.02	0.09	0.11	0.16	0.26	0.28	0.36	0.54	0.58	0.72
	PTT	0.05	0.06	0.05	0.1	0.11	0.1	0.14	0.21	0.11	0.17
*δ*_1_ = 0.75	Proposed	0.37	0.67	0.90	0.93	0.97	0.98	1.00	1.00	1.00	1.00
	Permutation	0.38	0.66	0.81	0.90	0.96	0.99	0.99	1.00	1.00	1.00
	SSF	0.04	0.21	0.37	0.50	0.81	0.86	0.91	0.96	0.97	0.98
	PTT	0.09	0.14	0.15	0.33	0.38	0.36	0.47	0.55	0.44	0.54
*δ*_1_ = 1.00	Proposed	0.61	0.92	0.97	1.00	1.00	1.00	1.00	1.00	1.00	1.00
	Permutation	0.57	0.89	0.95	0.99	1.00	1.00	1.00	1.00	1.00	1.00
	SSF	0.14	0.48	0.79	0.90	0.97	0.99	1.00	1.00	1.00	1.00
	PTT	0.08	0.23	0.42	0.43	0.54	0.62	0.77	0.77	0.79	0.85

**Table 2:** Table lists the empirical power of our proposed test and permutation test for Setting 2 with *δ*_2_ = 0.20, 0.30, 0.40, *δ*_1_ = *δ*_3_ = 0.00 and sample size ranging from 100 to 1000.

		Sample Size									
		100	200	300	400	500	600	700	800	900	1000
*δ*_2_ = 0.20	Proposed	0.28	0.46	0.66	0.79	0.86	0.95	0.95	0.97	0.98	0.99
	Permutation	0.27	0.43	0.59	0.74	0.86	0.94	0.94	0.98	1.00	1.00
	SSF	0.02	0.05	0.21	0.32	0.48	0.62	0.79	0.84	0.88	0.95
	PTT	0.04	0.03	0.04	0.08	0.11	0.14	0.12	0.09	0.16	0.26
*δ*_2_ = 0.30	Proposed	0.40	0.63	0.81	0.94	0.96	0.99	0.99	1.00	1.00	1.00
	Permutation	0.36	0.64	0.79	0.89	0.97	0.98	0.99	1.00	1.00	1.00
	SSF	0.03	0.13	0.35	0.52	0.72	0.85	0.91	0.97	0.99	1.00
	PTT	0.03	0.08	0.09	0.15	0.31	0.23	0.28	0.4	0.35	0.4
*δ*_2_ = 0.40	Proposed	0.73	0.98	1.00	1.00	1.00	1.00	1.00	1.00	1.00	1.00
	Permutation	0.78	0.98	1.00	1.00	1.00	1.00	1.00	1.00	1.00	1.00
	SSF	0.24	0.74	0.98	0.99	1.00	1.00	1.00	1.00	1.00	1.00
	PTT	0.11	0.16	0.18	0.38	0.39	0.52	0.56	0.59	0.81	0.89

**Table 3:** Table lists the empirical power of our proposed test and permutation test for Setting 3 with *δ*_1_, *δ*_2_ = (0.50, 0.20), (0.75, 0.30), (1.00, 0.40), *δ*_3_ = 0 and sample size ranging from 100 to 1000.

		Sample Size									
		100	200	300	400	500	600	700	800	900	1000
*δ*_1_ = 0.50	Proposed	0.35	0.51	0.74	0.86	0.91	0.95	0.97	0.98	1.00	1.00
*δ*_2_ = 0.20	SSF	0.04	0.15	0.29	0.41	0.57	0.72	0.85	0.89	0.91	0.96
	PTT	0.03	0.07	0.07	0.08	0.08	0.06	0.15	0.19	0.21	0.2
*δ*_1_ = 0.75	Proposed	0.42	0.70	0.86	0.96	0.99	1.00	1.00	1.00	1.00	1.00
*δ*_2_ = 0.30	SSF	0.05	0.26	0.46	0.64	0.79	0.93	0.94	0.95	1.00	1.00
	PTT	0.04	0.07	0.11	0.15	0.19	0.23	0.31	0.29	0.43	0.46
*δ*_1_ = 1.00	Proposed	0.72	0.98	1.00	1.00	1.00	1.00	1.00	1.00	1.00	1.00
*δ*_2_ = 0.40	SSF	0.25	0.72	0.97	0.99	1.00	1.00	1.00	1.00	1.00	1.00
	PTT	0.11	0.19	0.22	0.32	0.52	0.5	0.64	0.61	0.73	0.69

**Table 4:** Table lists the empirical power of our proposed test and permutation test for Setting 4 with *δ*_3_ = 0.50, 0.75, 1.00, *δ*_1_ = *δ*_2_ = 0.00 and sample size ranging from 100 to 1000.

		Sample Size									
		100	200	300	400	500	600	700	800	900	1000
*δ*_3_ = 0.50	Proposed	0.15	0.33	0.47	0.58	0.66	0.75	0.83	0.88	0.89	0.94
	SSF	0.01	0.04	0.07	0.16	0.18	0.28	0.35	0.47	0.57	0.64
	PTT	0.06	0.03	0.08	0.09	0.09	0.14	0.07	0.13	0.08	0.13
*δ*_3_ = 0.75	Proposed	0.35	0.61	0.73	0.84	0.92	0.95	0.99	1.00	1.00	1.00
	SSF	0.03	0.12	0.18	0.34	0.56	0.70	0.83	0.86	0.96	0.96
	PTT	0.01	0.07	0.06	0.07	0.09	0.12	0.13	0.18	0.18	0.24
*δ*_3_ = 1.00	Proposed	0.42	0.70	0.85	0.95	0.99	0.99	1.00	1.00	1.00	1.00
	SSF	0.07	0.20	0.52	0.76	0.82	0.92	0.98	0.98	1.00	1.00
	PTT	0.09	0.04	0.08	0.10	0.18	0.18	0.21	0.24	0.25	0.28

**Table 5:** Table lists the empirical sizes of the proposed test, permutation test, SSF, and PTT for *δ*_4_ = 0.00, 0.50, 1.00 and sample size ranging from 100 to 1000.

		Sample Size									
		100	200	300	400	500	600	700	800	900	1000
*δ*_4_ = 0.00	Proposed	0.04	0.07	0.06	0.06	0.05	0.06	0.06	0.07	0.06	0.05
	Permutation	0.04	0.08	0.05	0.08	0.06	0.05	0.06	0.04	0.07	0.06
	SSF	0.06	0.11	0.03	0.08	0.08	0.03	0.07	0.09	0.07	0.03
	PTT	0.03	0.05	0.02	0.02	0.03	0.02	0.12	0.09	0.08	0.06
*δ*_4_ = 0.50	Proposed	0.06	0.05	0.05	0.06	0.06	0.05	0.06	0.04	0.05	0.06
	Permutation	0.07	0.04	0.05	0.06	0.08	0.09	0.04	0.03	0.05	0.04
	SSF	0.06	0.05	0.07	0.06	0.07	0.08	0.07	0.04	0.04	0.07
	PTT	0.02	0.02	0.03	0.03	0.07	0.04	0.06	0.07	0.06	0.04
*δ*_4_ = 1.00	Proposed	0.07	0.06	0.07	0.06	0.05	0.05	0.06	0.06	0.06	0.05
	Permutation	0.04	0.06	0.03	0.05	0.05	0.04	0.03	0.02	0.04	0.04
	SSF	0.07	0.07	0.08	0.06	0.04	0.07	0.06	0.09	0.07	0.04
	PTT	0.03	0.04	0.03	0.05	0.05	0.04	0.06	0.05	0.05	0.08

**Table 6:** Table lists computational time (in hour) of running the simulation with 500 replications for our proposed test and the permutation test.

	Sample Size									
	100	200	300	400	500	600	700	800	900	1000
Proposed	0.01	0.03	0.04	0.06	0.07	0.09	0.10	0.12	0.14	0.16
Permutation	3.22	6.14	9.29	13.29	17.93	22.26	26.74	31.26	36.57	42.23

**Table 7:** Table lists the empirical size (*δ*_1_ = 0) and power (*δ*_1_ = 0.50, 0.75, 1.00) of our proposed test, SSF and PTT for Setting 6 with *δ*_2_ = *δ*_3_ = 0.00 and sample size ranging from 100 to 1000.

		Sample Size									
		100	200	300	400	500	600	700	800	900	1000
*δ*_1_ = 0.00	Proposed	0.08	0.04	0.06	0.06	0.06	0.08	0.10	0.07	0.06	0.07
	SSF	0.08	0.06	0.06	0.09	0.10	0.05	0.09	0.14	0.08	0.06
	PTT	0.02	0.1	0.04	0.08	0.11	0.05	0.16	0.12	0.13	0.06
*δ*_1_ = 0.50	Proposed	0.21	0.33	0.48	0.57	0.73	0.73	0.82	0.91	0.94	0.96
	SSF	0.01	0.05	0.10	0.17	0.29	0.32	0.46	0.48	0.63	0.72
	PTT	0.13	0.22	0.35	0.50	0.51	0.53	0.72	0.73	0.78	0.86
*δ*_1_ = 0.75	Proposed	0.66	0.89	0.98	1.00	1.00	1.00	1.00	1.00	1.00	1.00
	SSF	0.13	0.48	0.74	0.89	0.98	1.00	0.99	1.00	1.00	1.00
	PTT	0.16	0.32	0.41	0.43	0.66	0.67	0.73	0.85	0.85	0.89
*δ*_1_ = 1.00	Proposed	0.93	0.99	1.00	1.00	1.00	1.00	1.00	1.00	1.00	1.00
	SSF	0.47	0.93	0.99	1.00	1.00	1.00	1.00	1.00	1.00	1.00
	PTT	0.16	0.41	0.55	0.62	0.69	0.85	0.86	0.90	0.90	0.95

**Table 8:** Table lists the empirical size (*δ*_5_ = 0) and power (*δ*_5_ = 1.00, 2.00) of our proposed test, SSF and PTT for Setting 7.

		Sample Size									
		100	200	300	400	500	600	700	800	900	1000
*δ*_5_ = 0.00	Proposed	0.08	0.04	0.06	0.06	0.06	0.08	0.10	0.07	0.06	0.07
	SSF	0.01	0.01	0.00	0.00	0.00	0.01	0.01	0.01	0.00	0.00
	PTT	0.02	0.06	0.04	0.08	0.03	0.05	0.03	0.06	0.03	0.03
*δ*_5_ = 1.00	Proposed	0.21	0.33	0.48	0.57	0.73	0.73	0.82	0.91	0.94	0.96
	SSF	0.03	0.02	0.06	0.07	0.09	0.15	0.23	0.29	0.34	0.37
	PTT	0.01	0.05	0.01	0.02	0.04	0.02	0.04	0.04	0.08	0.06
*δ*_5_ = 2.00	Proposed	0.66	0.89	0.98	1.00	1.00	1.00	1.00	1.00	1.00	1.00
	SSF	0.07	0.24	0.46	0.63	0.76	0.87	0.91	0.98	0.97	0.99
	PTT	0.02	0.04	0.02	0.09	0.03	0.06	0.07	0.10	0.06	0.06

## Footnotes

1. The *m*th order Sobolev space is defined as $H^{\langle 1\rangle }=\left\{\eta_{1}\in L_{2}[0,1]|\eta_{1}^{\left(k\right)}\text{ is absolutely continuous for }k=0,1,\ldots ,m-1\right\}$.

2. Data used in preparation of this article were obtained from the Alzheimer’s Disease Neuroimaging Initiative (ADNI) database (adni.loni.usc.edu). As such, the investigators within the ADNI contributed to the design and implementation of ADNI and/or provided data but did not participate in analysis or writing of this report. A complete listing of ADNI investigators can be found at http://adni.loni.usc.edu/wp-content/uploads/how_to_apply/ADNI_Acknowledgement_List.pdf

## Appendix A. Proof of Main Results

In this section, we present main proofs of the theorems and lemmas in the main text.

### A.1. Notation Clarification

We rewrite (16) as

$${\hat{f}}_{11}=K_{11}M^{-1}\left(I_{ns}-S{\left(S^{T}M^{-1}S\right)}^{-1}S^{T}M^{-1}\right)y,$$

where

$$S=\left[\begin{array}{cc} I_{n}⊗1_{w_{0}} & 0\\ 0 & I_{n}⊗1_{w_{1}} \end{array}\right]\left[\begin{array}{cc} 1_{n} & 1_{n}\\ 1_{n} & 0 \end{array}\right],K_{11}=\frac{1}{2}\left[\begin{array}{cc} K_{1}^{\langle 1\rangle } & -K_{1}^{\langle 1\rangle }\\ -K_{1}^{\langle 1\rangle } & K_{1}^{\langle 1\rangle } \end{array}\right]$$

and

$$M=\left[\begin{array}{cc} I_{n}⊗1_{w_{0}} & 0\\ 0 & I_{n}⊗1_{w_{1}} \end{array}\right]\left(\frac{\theta_{10}}{2}\left[\begin{array}{cc} K_{1}^{\langle 1\rangle } & K_{1}^{\langle 1\rangle }\\ K_{1}^{\langle 1\rangle } & K_{1}^{\langle 1\rangle } \end{array}\right]+\frac{\theta_{11}}{2}\left[\begin{array}{cc} K_{1}^{\langle 1\rangle } & -K_{1}^{\langle 1\rangle }\\ -K_{1}^{\langle 1\rangle } & K_{1}^{\langle 1\rangle } \end{array}\right]+\lambda I_{2n}\right)\left[\begin{array}{cc} I_{n}⊗1_{w_{0}}^{T} & 0\\ 0 & I_{n}⊗1_{w_{1}}^{T} \end{array}\right],$$

$K_{1}^{\langle 1\rangle }$ is the kernel matrix of $H_{1}^{\langle 1\rangle }$ with (*i*, *i*′)th entry as $\frac{1}{n}K_{1}^{\langle 1\rangle }\left(x_{i}^{\langle 1\rangle },x_{i^{\prime }}^{\langle 1\rangle }\right)$, *w*_0_ is the number of subjects in control group, *w*_1_ is the number of subjects in case group, and ⊗ denotes the Kronecker product. Based on Chapter A.3 in Gu (2013), we set $\theta_{10}^{-1}\propto \text{Tr}\left(K_{10}\right)$ and $\theta_{11}^{-1}\propto \text{Tr}\left(K_{11}\right)$ with *θ*_10_ + *θ*_11_ = 1.

In the following theoretical derivation, we only focus on the case with *s* = 2, i.e. *w*_0_ = *w*_1_ = 1. If we have *s* > 2 subjects, the proof can be easily generalized to this situation by replacing Equation (15) by the penalized weighted least squares; see Section 3.2.4 in Gu (2013).

### A.2. Proofs for Section 3

#### A.2.1. Preliminary

We identify a sequence model that is equivalent to our nonparametric model (1) with SSANOVA decomposition in Equation (2). Let ${\left\{\rho_{i},\phi_{i}\right\}}_{i=1}^{\infty }$ be pairs of eigenvalue and eigenfunction in $H^{\langle 1\rangle }$ and ${\left\{\nu_{j},\psi_{j}\right\}}_{j=1}^{2}$ be pairs of eigenvalue and eigenfunction in $H^{\langle 2\rangle }$. In the tensor product space $H=H^{\langle 1\rangle }⊗H^{\langle 2\rangle }$, as shown in Lin (2000), eigenvalues and eigenfunctions are {*μ*_*i*_*ν*_*j*_, *ϕ*_*i*_*ψ*_*j*_}_*i*=1,…,∞, *j*=1,2_. Model (1) is equivalent to a sequence model

(30)
$$z_{ij}=\theta_{ij}+\omega_{ij},$$

where $\theta_{ij}=\frac{1}{2}\sum_{x^{\langle 2\rangle }=0}^{1}\int_{X_{1}}f\left(x_{i}^{\langle 1\rangle },x_{j}^{\langle 2\rangle }\right)\phi_{i}\left(x^{\langle 1\rangle }\right)\psi_{j}\left(x^{\langle 2\rangle }\right)d\omega \left(x^{\langle 1\rangle }\right)$ are the basis expansion coefficients, the random noise *ω*_*ij*_ is mean zero and variance *σ*^2^/*n*. The space $E=\left\{f\in H:\|f{\|}_{H}<1\right\}$ in Equation (30) is equivalent to $E=\left\{\sum_{i=1}^{\infty }\sum_{j=1}^{2}\frac{\theta_{ij}^{2}}{\left(\mu_{i}\nu_{j}\right)}\leq 1\right\}$. The hypothesis in Equation (18) is equivalent to the hypothesis

$$H_{0}:\theta_{i2}=0\text{ for }i=2,\ldots ,n.$$

Let ***θ***_11_ = (*θ*_22_, *θ*_32_, …, *θ*_*n*2_)^*T*^, and $E_{11}=\left\{\theta_{11}|\sum_{i=2}^{n}\frac{\theta_{i2}^{2}}{\left(\mu_{i}\nu_{2}\right)}\leq 1\right\}$. Consider a local alternative $H_{1n}:\theta_{11}\in E_{11}$ with ∥***θ***_11_∥_2_ ≥ *d*_*n*_, where *d*_*n*_ represents a generic distinguishable rate. The total error of a generic testing rule *ϕ*_*n*_ under distinguishable rate *d*_*n*_ can be rewritten as

(31)
$$\text{pseudo.risk}\left(\phi_{n},d_{n}\right)=E_{H_{0}}\left\{\phi_{n}|H_{0}\text{ is true}\right\}+\underset{\begin{array}{l} \theta_{11}\in E_{11}\\ {\left\|\theta_{11}\right\|}_{2}\geq d_{n} \end{array}}{\text{sup}}E\left\{1-\phi_{n}|H_{1}\text{ is true}\right\}.$$

Equation (31) is consistent with the testing error defined by Ingster (1993), Wei and Wainwright (2020). For the simplicity of description, we order the axis length ${\left\{\left(\mu_{i}\nu_{2}\right)\right\}}_{i=2}^{\infty }$ from the smallest to the largest as ${\left\{\rho_{p}\right\}}_{p=1}^{\infty }$. Next we introduce a lemma to give a low bound of the minimum pseudo risk.

**Lemma 11** *For every set C and probability measure Q supported on* $C\cap B^{c}\left(d_{n}\right)$, *we have*

$$\underset{\phi_{n}}{\text{inf }}\text{pseudo.risk}\left(\phi_{n},d_{n}\right)\geq 1-\frac{1}{2}\sqrt{E_{\eta ,\eta^{\prime }}\text{ exp}\left(\frac{\langle \eta ,\eta^{\prime }\rangle }{\sigma^{2}}\right)-1}$$

*where* $E_{\eta ,\eta^{\prime }}$ *denotes expectation with respect to an i.i.d. pair η*, $\eta^{\prime }\sim \mathbb{Q}$

The proof of this lemma directly follows Lemma 3 in Wei and Wainwright (2020).

#### A.2.2. Proof of Lemma 4

**Proof** As shown in Lemma 11, we have

(32)
$$\underset{\phi_{n}}{\text{inf }}\text{pseudo.risk}\left(\phi_{n},d_{n}\right)\geq 1-\frac{1}{2}\sqrt{E_{\eta ,\eta^{\prime }}\text{ exp}\left(\frac{\langle \eta ,\eta^{\prime }\rangle }{\sigma^{2}}\right)-1}$$

Next we show that if $\delta^{2}\leq \frac{\sqrt{k_{B}(\delta )}\sigma^{2}}{4}$, we have the last term in Equation (32) larger than 1/2. Let $\theta_{b}=\frac{\delta }{\sqrt{k}}\sum_{i=1}^{k}b_{i}e_{i}$ where *e*_*i*_ is the standard basis vector with *i*th coordinate as one. We consider Q as the uniform distribution on {*θ*_*b*_, *b* ∈ {−1, 1}^*k*^}. The expectation in the last term of Equation (32) can be written as

$$E_{n\eta ,\eta^{\prime }}\text{ exp}\left(\frac{n\langle \eta ,\eta^{\prime }\rangle }{\sigma^{2}}\right)=\frac{1}{2^{k}}\sum_{b,b^{\prime }}\text{exp}\left(\frac{n\theta_{b}^{T}\theta_{b^{\prime }}}{\sigma^{2}}\right)=\frac{1}{2^{k}}\sum_{b,b^{\prime }}\text{exp}\left(\frac{n\delta^{2}\sum_{i=1}^{k}b_{i}b_{i}^{\prime }}{k\sigma^{2}}\right)\;=\frac{1}{2^{k}}{\left(\text{exp}\left(\frac{n\delta^{2}}{k\sigma^{2}}\right)+\text{exp}\left(-\frac{n\delta^{2}}{k\sigma^{2}}\right)\right)}^{k}\;\overset{\left(i\right)}{\leq }{\left(1+\frac{n^{2}\delta^{4}}{k^{2}\sigma^{4}}\right)}^{k}\;\overset{\left(ii\right)}{\leq }\text{exp}\left(\frac{n^{2}\delta^{4}}{k\sigma^{4}}\right),$$

where (i) is due to that $\frac{1}{2}(\text{exp}(x)+\text{exp}(-x))\leq 1+x^{2}$ for |*x*| ≤ 1/2 and (ii) is due to that 1+*x* ≤ *e*^*x*^. Thus for any $\delta^{4}\leq \frac{k\sigma^{4}}{16n^{2}}$, we have

$$\underset{\phi_{n}}{\text{inf }}\text{pseudo.risk}\left(\phi_{n},d_{n}\right)\geq 1-\frac{1}{2}\sqrt{e^{1/16}-1}\geq 1/2.$$

By the definition of *r*_*B*_, we have *pseudo.risk*(*ϕ*_*n*_, *d*_*n*_) > 1/2 for all *d*_*n*_ ≤ *r*_*B*_. ■

#### A.2.3. Proof of Lemma 5

**Proof** We show that $b_{k,2}\left(E_{11}\right)$ is bounded below by $\sqrt{\rho_{k+1}}$. It is sufficient to show that $E_{11}$ contains a *l*_2_ ball centered at *f*_11_ = 0 with radius $\sqrt{\rho_{k+1}}$. For any $v\in E_{11}$ with $\|v{\|}_{2}\leq \sqrt{\rho_{k+1}}$, we have

$$b_{2,k}\overset{\left(i\right)}{\leq }\overset{k+1}{\underset{i=1}{\sum }}\frac{v_{i}^{2}}{\rho_{i}}\overset{\left(ii\right)}{\leq }\frac{1}{\mu_{k+1}}\overset{k+1}{\underset{i=1}{\sum }}v_{i}^{2},$$

where inequality (i) holds by set the (*k* + 1)-dimensional subspace spaned by the eigenvectors corresponding to the first (*k* + 1) largest eigenvalues; inequality (ii) holds by the decreasing order of the eigenvalues, i.e., *ρ*_1_ ≥ *ρ*_2_ ≥ … *ρ*_*k*+1_.

Recall that the definition of the Bernstein lower critical dimension is $k_{B}(\delta )=\text{arg }{\text{max}}_{k}\left\{b_{k-1,2}^{2}\left(E_{11}\right)\geq \delta^{2}\right\}$, we have

$$k_{B}(\delta )\geq \underset{k}{\text{arg max}}\left\{\sqrt{\rho_{k}}\geq \delta \right\}.$$

#### A.2.4. Proof of Theorem 6

**Proof** By Lemme 4, we have

$$d_{n}\leq \text{sup}\left\{\delta :k_{B}(\delta )\geq 16n^{2}\delta^{4}\right\}.$$

We plug in the lower bound of *k*_*B*_(*δ*) in Lemma 5. Then we have

(33)
$$d_{n}\leq \text{sup}\left\{\delta :\underset{k}{\text{arg max}}\left\{\sqrt{\rho_{k}}\geq \delta \right\}\geq 16n^{2}\delta^{4}\right\}.$$

The eigenvalues have polynomial decay rate i.e., *ρ*_*p*_ ≍ *p*^−2*m*^, and consequently, $\text{arg }{\text{max}}_{k}\left\{\sqrt{\rho_{k}}\geq \delta \right\}≍\delta^{-1/m}$. Plugging this into Equation (33), it is easy to see that the supremum on the right hand side has an order $n^{-\frac{2m}{4m+1}}$. Proof is thus completed. ■

### A.3. Proof of Theorem 7

Before deriving the proof of Theorem 7, we first state Lemma 12, Lemma 13, Lemma 14, and Lemma 15, which are used in the proof of Theorem 7. The proof of these auxiliary lemmas is referred to the Supplementary.

#### A.3.1. Some Auxiliary Lemmas

Lemma 12 shows the projection of ***f***_10_ on $H_{11}\cap H_{\text{model}}$ is zero. This result indicates our test statistic does not depend on the nuisance parameter ***f***_10_.

**Lemma 12** *The quantity, K*_11_*M*^−1^(*I*_*n*_ − *S*(*S*^*T*^ *M*^−1^*S*)^−1^*S*^*T*^ *M*^−1^)***f***_10_, *equals to zero*.

The next two lemmas show the equivalence of *τ*_*λ*_ and ${\hat{\tau }}_{\lambda }$ under the quasi-uniform design and uniform design.

**Lemma 13** *If x*^〈1〉^ *follows the quasi-uniform random design, for any* $\lambda =\frac{1}{n^{1-c}}$, *m* > 3/2, *and any δ*, *c* > 0, *we have*

$$P\left({\hat{\tau }}_{\lambda }≍\tau_{\lambda }\right)\geq 1-\left(n^{\frac{2}{2m-1}-2\delta }+n^{\frac{1}{2m-1}}\right)\text{ exp}\left\{-cn^{\frac{2m-3}{2m-1}+2\delta }\right\},$$

*where τ*_*λ*_ = max{*i* | *μ*_*i*_ ≥ *λ*} *and* ${\hat{\tau }}_{\lambda }=\text{max}\left\{i|{\hat{\mu }}_{i}\geq \lambda \right\}$.

**Lemma 14** *If x*^〈1〉^ *follows the uniform fixed design condition, for m* > 1/2 *and λ* > 0*, we have*

$${\hat{\tau }}_{\lambda }≍\tau_{\lambda }.$$

In the following lemma, we bound Tr(Δ) by a function of ${\hat{\tau }}_{\lambda }$. This result is essential in deriving the asymptotic distribution of *T*_*n*,*λ*_.

**Lemma 15** *For* $\Delta =M^{-1}K_{11}^{2}M^{-1}$ *defined in Theorem 7, we have*

(34)
$$\frac{4{\hat{\tau }}_{\lambda }}{9}\leq \text{Tr}(\Delta )\leq \frac{4}{{\left(1-\theta_{d}\right)}^{2}}\left({\hat{\tau }}_{\lambda }+\frac{1}{2\lambda }\overset{n}{\underset{i={\hat{\tau }}_{\lambda }+1}{\sum }}{\hat{\mu }}_{i}\right).$$

#### A.3.2. Proof of Theorem 7

**Proof** For simplicity, we suppose *σ*^2^ = 1. We define the three terms on the right-hand side of Equation (26) as *T*_1_, *T*_2_ and *T*_3_, i.e.,

$$T_{1}=\frac{1}{n}\epsilon^{T}\Delta \epsilon ,\;T_{2}=\frac{1}{n}\epsilon^{T}M^{-1}S{\left(S^{T}M^{-1}S\right)}^{-1}S^{T}\Delta S{\left(S^{T}M^{-1}S\right)}^{-1}S^{T}M^{-1}\epsilon ,\;T_{3}=\frac{1}{n}\epsilon^{T}M^{-1}S{\left(S^{T}M^{-1}S\right)}^{-1}S^{T}\Delta \epsilon .$$

We now show *T*_2_ and *T*_3_ are in smaller order compared to *T*_1_. First, we analyze the second term *T*_2_ in Equation (26). We have

$$E\left[T_{2}\right]=\frac{1}{n}E\left[\epsilon^{T}M^{-1}S{\left(S^{T}M^{-1}S\right)}^{-1}S^{T}\Delta S{\left(S^{T}M^{-1}S\right)}^{-1}S^{T}M^{-1}\epsilon \right]\;=\frac{1}{n}\text{Tr}\left(M^{-1}S{\left(S^{T}M^{-1}S\right)}^{-1}S^{T}\Delta S{\left(S^{T}M^{-1}S\right)}^{-1}S^{T}M^{-1}\right)\;\leq \frac{2}{n}\lambda_{\text{max}}(\Delta )\lambda_{\text{max}}\left(M^{-1}S{\left(S^{T}M^{-1}S\right)}^{-1}S^{T}S{\left(S^{T}M^{-1}S\right)}^{-1}S^{T}M^{-1}\right)\;\leq \frac{2}{n}\lambda_{\text{max}}(\Delta ),$$

where *λ*_max_ denotes the largest eigenvalue. Since all eigenvalues of Δ are less than 1, we have $E\left[T_{2}\right]\leq \frac{2}{n}$. Analogously, we can derive the variance inequality of *T*_2_. Combining the results together and using the Chebyshev inequality, we have

(35)
$$T_{2}=O_{p}\left(\frac{1}{n}\right).$$

Second, we analyze the third term *T*_3_ in Equation (26). We apply the Cauchy-Schwarz inequality and have

(36)
$$\left|T_{3}\right|\leq \sqrt{T_{2}}\sqrt{T_{1}}.$$

Finally, we derive the magnitude of *T*_1_. We first consider the testing consistency of *T*_1_ conditional on *X*. Denote $E_{\epsilon }$ as the expectation with respect to *ϵ*, and define $Var_{\epsilon }$ as the variance with respect to *ϵ*. Note that

$$E_{\epsilon }\left[\epsilon^{T}\Delta \epsilon \right]=\text{Tr}(\Delta ),\;Var_{\epsilon }\left[\epsilon^{T}\Delta \epsilon \right]=2\text{Tr}\left(\Delta^{2}\right).$$

Let $Z=\left(\epsilon^{T}\Delta \epsilon -\text{Tr}(\Delta )\right)/\sqrt{2\text{Tr}\left(\Delta^{2}\right)}$ and *t* ∈ (−1/2, 1/2). Then the log-characteristic function of *Z* can be written as

(37)
$$\text{log }E_{\epsilon }[\text{exp}(itZ)]\;=\text{log }E_{\epsilon }\left[\text{exp}\left(it\epsilon^{T}\Delta \epsilon /\sqrt{2\text{Tr}\left(\Delta^{2}\right)}\right)\right]-it\text{Tr}(\Delta )/\sqrt{2\text{Tr}\left(\Delta^{2}\right)}\;=-\frac{1}{2}\text{log det}\left\{I_{2n}-2it\Delta /\sqrt{2\text{Tr}\left(\Delta^{2}\right)}\right\}-it\text{Tr}(\Delta )/\sqrt{2\text{Tr}\left(\Delta^{2}\right)}.$$

Through Taylor expansion, one has

(38)
$$-\frac{1}{2}\text{log det}\left\{I_{2n}-2it\Delta /\sqrt{2\text{Tr}\left(\Delta^{2}\right)}\right\}\;=it\frac{\text{Tr}(\Delta )}{\sqrt{2\text{Tr}\left(\Delta^{2}\right)}}-t^{2}\frac{\text{Tr}\left(\Delta^{2}\right)}{2\text{Tr}\left(\Delta^{2}\right)}+O\left(t^{3}\frac{\text{Tr}\left(\Delta^{3}\right)}{{\left[\text{Tr}\left(\Delta^{2}\right)\right]}^{3/2}}\right).$$

Combining Equations (37) and (38), we have

(39)
$$\text{log }E_{\epsilon }[\text{exp}(itZ)]=-\frac{t^{2}}{2}+O\left(t^{3}\frac{\text{Tr}\left(\Delta^{3}\right)}{{\left[\text{Tr}\left(\Delta^{2}\right)\right]}^{3/2}}\right).$$

Since all eigenvalues of Δ are less than 1, we have $\frac{\text{Tr}\left(\Delta^{3}\right)}{\text{Tr}\left(\Delta^{2}\right)}\leq 1$. Analogous to (S.11), we have

(40)
$$\text{Tr}\left(\Delta^{2}\right)\geq \frac{16}{81}{\hat{\tau }}_{\lambda }.$$

Under the quasi-uniform design, we have Tr(Δ^2^) → ∞ as *λ* → 0 with probability approaching 1 by Lemma 13 and Equation (40). Hence, the second term on the right-hand side of Equation (39) is *o*_*p*_(1). We thus conclude that

$$E_{\epsilon }[\text{exp}(itZ)]\overset{P}{\to }\text{exp}\left(-\frac{t^{2}}{2}\right).$$

Next, we show that

$$E[\text{exp}(itZ)]=E_{X}\left[E_{\epsilon }[\text{exp}(itZ)]\right]\to \text{exp}\left(-t^{2}/2\right)$$

for $t\in \left(-\frac{1}{2},\frac{1}{2}\right)$. If not, there exists a subsequence of r.v $X_{nk}^{\langle 1\rangle }$, such that for ∀*ε* > 0, $\left|E_{X_{nk}^{\langle 1\rangle }}E_{\epsilon }\text{ exp}(itZ)-\text{exp}\left(-t^{2}/2\right)\right|>\varepsilon$. On the other hand, since $E_{\epsilon }\text{ exp}\left(itZ\left(X_{nk}^{\langle 1\rangle }\right)\right)\overset{P}{\to }\text{exp}\left(-t^{2}/2\right)$, which is bounded, there exists a sub-sub sequence $\left\{X_{n_{kl}}^{\langle 1\rangle }\right\}$, such that $E_{\epsilon }\text{ exp}\left(itZ\left(X_{n_{kl}}^{\langle 1\rangle }\right)\right)\overset{a.s}{\to }\text{exp}\left(-t^{2}/2\right)$. Then by dominate convergence theorem, $E_{X_{n_{kl}}^{\langle 1\rangle }}E_{\epsilon }\text{ exp}(itZ)\to \text{exp}\left(-t^{2}/2\right)$, which is a contradiction. Under the uniform design, we can easily obtain $E[\text{exp}(itZ)]\to \text{exp}\left(-\frac{t^{2}}{2}\right)$ by Lemma 14 and Equation (40).

Thus *Z* is asymptotically normally distributed, and

(41)
$$\frac{T_{1}-\text{Tr}(\Delta )/n}{\sqrt{2\text{Tr}\left(\Delta^{2}\right)/n^{2}}}\overset{d}{\to }N(0,1).$$

Combining (35), (36) and (41), the theorem follows. ■

### A.4. Proof of Theorem 8

**Proof** Under the alternative hypothesis, the statistic *T*_*n*,*λ*_ in Equation (26) can be decomposed into three terms as follows

(42)
$$T_{n,\lambda }=\frac{1}{n}\|H\epsilon {\|}_{2}^{2}+\frac{1}{n}{\left\|Hf_{11}\right\|}_{2}^{2}+\frac{2}{n}f_{11}^{T}H^{T}H\epsilon .$$

where *H* = *θ*_11_*K*_11_*M*^−1^(*I* − *S*(*S*^*T*^*M*^−1^*S*)^−1^*S*^*T*^*M*^−1^). Let $W_{1}=\frac{1}{n}\|H\epsilon {\|}_{2}^{2}$, $W_{2}=\frac{1}{n}{\left\|Hf_{11}\right\|}_{2}^{2}$, and $W_{3}=\frac{2}{n}f_{11}^{T}H^{T}H\epsilon$ denote corresponding three terms on the right-hand side of Equation (42).

We now derive a lower bound for *W*_2_. By Lemma S.1, we have

(43)
$$\frac{1}{n}{\left\|Hf_{11}-f_{11}\right\|}_{2}^{2}\leq \frac{1}{n}{\left\|Hf_{11}-f_{11}\right\|}_{2}^{2}+\frac{1}{n}{\left\|Hf_{10}-f_{10}\right\|}_{2}^{2}\;=\frac{1}{n}{\left\|Hf_{10}+Hf_{11}-f_{10}-f_{11}\right\|}_{2}^{2}\;={\left\|\tilde{g}*-g*\right\|}_{n}^{2}\leq c\lambda .$$

Let $c^{\prime }=\sqrt{c}$, we consider the distinguishable rate

(44)
$$\frac{1}{n}{\left\|f_{11}\right\|}_{2}^{2}={\left\|f_{11}\right\|}_{n}^{2}>c^{\prime 2}d_{n}^{2}=c\left(\lambda +\sigma_{n,\lambda }\right).$$

where the inequality is satisfied since ∥ · ∥_*n*_ dominates ∥ · ∥_2_ by Lemma S.2. The lower bound of *W*_2_ is thus,

(45)
$$W_{2}=\frac{1}{n}{\left\|Hf_{11}\right\|}_{2}^{2}=\frac{1}{n}{\left\|f_{11}\right\|}_{2}^{2}-\frac{1}{n}{\left\|f_{11}-Hf_{11}\right\|}_{2}^{2}\geq cd_{n}^{2}-c\lambda \geq c\sigma_{n,\lambda }.$$

where the first inequality is obtained by (43) and the second inequality is obtained through plugging in Equation (44).

For the third term *W*_3_, it is seen that $EW_{3}=0$. It is easy to verify that the eigenvalues of *HH*^*T*^ are all less than 1. Moreover,

$$EW_{3}^{2}=\frac{4}{n^{2}}E\left[f_{11}^{T}H^{T}H\epsilon \epsilon^{T}H^{T}Hf_{11}\right]=\frac{4}{n^{2}}{\left(Hf_{11}\right)}^{T}HH^{T}\left(Hf_{11}\right)\;\leq \frac{4}{n^{2}}{\left(Hf_{11}\right)}^{T}\left(Hf_{11}\right)=\frac{4}{n}W_{2}.$$

By the Chebyshev’s inequality, for any *ϵ* > 0, we have

$$\mathbb{P}\left(\left|W_{3}\right|\geq \frac{2\epsilon^{-\frac{1}{2}}W_{2}^{\frac{1}{2}}}{\sqrt{n}}\right)\leq \frac{nEW_{3}^{2}}{4\epsilon^{-1}W_{2}}\leq \epsilon .$$

Consequently, there exists an *n*_0_, for any *n* > *n*_0_, we have

(46)
$$\mathbb{P}\left\{\left|W_{3}\right|>\frac{1}{2}W_{2}\right\}\leq \mathbb{P}\left(\left|W_{3}\right|\geq \frac{2\epsilon^{-\frac{1}{2}}W_{2}^{\frac{1}{2}}}{\sqrt{n}}\right)\leq \epsilon .$$

Now, we are ready to prove our theorem. By the triangle inequality, we have

(47)
$$\left|\frac{W_{1}-\mu_{n,\lambda }}{\sigma_{n,\lambda }}+\frac{W_{2}+W_{3}}{\sigma_{n,\lambda }}\right|\geq \left|\frac{W_{2}+W_{3}}{\sigma_{n,\lambda }}\right|-\left|\frac{W_{1}-\mu_{n,\lambda }}{\sigma_{n,\lambda }}\right|$$

(48)
$$\geq \left|\frac{W_{2}}{\sigma_{n,\lambda }}\right|-\left|\frac{W_{3}}{\sigma_{n,\lambda }}\right|-\left|\frac{W_{1}-\mu_{n,\lambda }}{\sigma_{n,\lambda }}\right|.$$

If $\frac{\left|W_{1}-\mu_{n,\lambda }\right|}{\sigma_{n,\lambda }}\leq C_{\epsilon }$, and $\left|W_{3}\right|\leq \frac{1}{2}W_{2}$ hold, in view of (47) and Equation (45), we have

$$\left|\frac{W_{1}-\mu_{n,\lambda }}{\sigma_{n,\lambda }}+\frac{W_{2}+W_{3}}{\sigma_{n,\lambda }}\right|\geq \frac{1}{2}c-C_{\epsilon }.$$

Noting that *W*_1_ is identical to Equation (26), by Theorem 7, we have $\frac{\left|W_{1}-\mu_{n,\lambda }\right|}{\sigma_{n,\lambda }}=O_{p}(1)$. That is for any *C*_*ϵ*_ > 0, there exists an *s*, for any *n* > *s*, we have

(49)
$$\mathbb{P}\left(\frac{\left|W_{1}-\mu_{n,\lambda }\right|}{\sigma_{n,\lambda }}>C_{\epsilon }\right)\leq \epsilon .$$

Setting $c\geq 2\left(C_{\epsilon }+z_{1-\frac{\alpha }{2}}\right)$ and *N* = max(*n*_0_, *s*), for any *n* > *N*, we have

$$\mathbb{P}\left(\phi_{n,\lambda }=1\right)=\mathbb{P}\left\{\frac{\left|W_{1}+W_{2}+W_{3}-\mu_{n,\lambda }\right|}{\sigma_{n,\lambda }}\geq z_{1-\frac{\alpha }{2}}\right\}\;\geq \mathbb{P}\left\{\frac{\left|W_{1}-\mu_{n,\lambda }\right|}{\sigma_{n,\lambda }}\leq C_{\epsilon },\left|W_{3}\right|\leq \frac{1}{2}W_{2}\right\}\;\geq 1-\mathbb{P}\left\{\frac{\left|W_{1}-\mu_{n,\lambda }\right|}{\sigma_{n,\lambda }}>C_{\epsilon }\right\}-\mathbb{P}\left\{\left|W_{3}\right|>\frac{1}{2}W_{2}\right\}\;\geq 1-2\epsilon .$$

where the second inequality is due to the Boole’s inequality (Casella and Berger, 2002) and the last inequality is obtained by combining Equation (45) and Equation (49). Thus, we have

$$\underset{H_{1}^{*}}{\text{sup }}E\left(1-\phi_{n,\lambda }|H_{1}^{*}\text{ is true}\right)<\delta ,$$

where $H_{1}^{*}=\left\{f|f\in H_{\text{model}}^{\infty }\text{ and }{\left\|f_{11}\right\|}_{2}\geq C_{\delta }\sqrt{\lambda +\sigma_{n,\lambda }}≜d_{n}\right\}$. ■
