# Supplementary material for: Minimax Nonparametric Parallelism Test
Source: J Mach Learn Res. Author manuscript; Available in PMC 2024 May 10. (PMC11086968)
Supplement: 1 [file NIHMS1641658-supplement-1.pdf]

Supplementary

Supplement to
Minimax Nonparametric Parallelism Test

This document contains some auxiliary lemmas, the proofs of Corollary 9 and Corollary 10 as well as the proofs of Lemma 12, Lemma 13, Lemma 14, and Lemma 15 stated in Appendix.

- Section S.1 includes some auxiliary lemmas in proving Theorem 8.
- Section S.2 includes the proof of Corollary 9 and Corollary 10.
- Section S.3 includes the proof of Lemma 12, Lemma 13, Lemma 14, Lemma 15, Lemma S.1, Lemma S.2, and Lemma S.4.

S.1. Some Auxiliary Lemmas in Proving Theorem 8

We first introduce several notations and lemmas and then start the main proof of Theorem 8.
Let $g^* = f_{10} + f_{11}$ and its estimator as

$$\tilde{\boldsymbol{g}}^* = R[M^{-1} - M^{-1}S(S^TM^{-1}S)^{-1}S^TM^{-1}]\boldsymbol{g}^*. \tag{S.1}$$

Lemma S.1 *If $\|f\|_{\mathcal{H}} < 1$ for any $f \in \mathcal{H}$, as $n \to \infty$, $\lambda \to 0$ and $\lambda \geq n^{-1}$, we have*

$$||\tilde{g}^* - g^*||_n^2 \leq c\lambda,$$

where c is a constant, $||\cdot||_n$ is the empirical norm.

In the following Lemma S.2, we discuss the relationship between the empirical norm and L_2 norm. Recall the definition of empirical norm and L_2 norm are as follows:

$$||f||_n^2 = \frac{1}{2n}\sum_{j=1}^{2}\sum_{i=1}^{n} f^2(x_i^{\langle 1\rangle}, x_j^{\langle 2\rangle}) \quad \text{and} \quad ||f||_2^2 = \sum_{x^{\langle 2\rangle}=0}^{1}\int_0^1 f^2(x^{\langle 1\rangle}, x^{\langle 2\rangle})d\omega_1.$$

Lemma S.2 *Under the quasi-uniform design or the uniform design, for $f : \mathcal{X}_1 \times \mathcal{X}_2 \to \mathbb{R}$ and a positive constant c, we have*

$$||f||_2 \leq c||f||_n,$$

i.e. the empirical norm of f dominates the L_2 norm.

S.2. Proofs of Corollary 9 and Corollary 10

In order to find the optimal distinguishable rate, we need to bound the tail sum of the eigenvalues of the empirical kernel matrix. We state the following two lemmas which give upper bounds for the tail sum of the eigenvalues of the empirical kernel matrix under the quasi-uniform design and the uniform design respectively.

Lemma S.3 *(Liu et al. (2019)) If $1/n < \lambda \to 0$ and the quasi-uniform design is satisfied, then with probability at least $1 - 4e^{-\tau_\lambda}$,*

$$\sum_{i=\hat{\tau}_\lambda+1}^{n} \hat{\mu}_i \leq C\tau_\lambda \mu_{\tau_\lambda},$$

where $C > 0$ is an absolute constant.

Lemma S.4 *If $\lambda > 0$ and the uniform design is satisfied, we have*

$$\sum_{i=\hat{\tau}_\lambda+1}^{n} \hat{\mu}_i \leq C\tau_\lambda \mu_{\tau_\lambda},$$

where $C > 0$ is an absolute constant.

Now we start the main proof of Corollary 9 and Corollary 10. The distinguishable rate is

$$d_n = \sqrt{\lambda + \sigma_{n,\lambda}},$$

where $\sigma_{n,\lambda}^2 = 2\theta_{11}^4 \sigma^4 \operatorname{Tr}(\Delta^2)/n^2$. We now derive the order of $\sigma_{n,\lambda}^2$. Since the eigenvalues of Δ are less than 1, and by Lemma 15, we have

$$\operatorname{Tr}(\Delta^2) \leq \operatorname{Tr}(\Delta) \leq \frac{4}{(1-\theta_d)^2}(\hat{\tau}_\lambda + \frac{1}{2\lambda} \sum_{i=\hat{\tau}_\lambda+1}^{n} \hat{\mu}_i).$$

Proof Under the quasi-uniform design, applying Lemma A.1, we have

$$\operatorname{Tr}(\Delta^2) \lesssim \frac{4}{(1-\theta_d)^2}(\hat{\tau}_\lambda + \frac{1}{2\lambda}\lambda\tau_\lambda)$$

with probability at least $1 - 4e^{-\tau_\lambda}$. Combining the lower bound of $\operatorname{Tr}(\Delta^2)$ in Equation (40) and Lemma S.3, we have

$$\operatorname{Tr}(\Delta^2) \asymp \tau_\lambda, \tag{S.2}$$

with probability at least $1 - 4e^{-\tau_\lambda} - (n^{\frac{2}{2m-1}-2\epsilon} + n^{\frac{1}{2m-1}}) \exp\{-cn^{\frac{2m-3}{2m-1}+2\epsilon}\}$. Similarly, we have Equation (S.2) satisfied under the uniform design by applying Lemma 14 and Lemma S.4.

Using Equation (S.2), we have

$$\sigma_{n,\lambda}^2 \asymp \lambda^{-\frac{1}{2m}} n^{-2} \asymp \tau_\lambda n^{-2}. \tag{S.3}$$

By the Cauchy-Schwartz inequality, the distinguishable rate $d_n = \sqrt{\lambda + \sigma_{n,\lambda}}$ is minimized when $\lambda \asymp \sigma_{n,\lambda}$, i.e.,

$$\lambda \asymp n^{-4m/(4m+1)}.$$

Thus we have the minimum distinguishable rate

$$d_n^* \asymp n^{-2m/(4m+1)}.$$

By Lemma S.2, this optimal distinguishable rate is achieved in the sense of L_2 norm. ■

S.3. Proofs of Auxiliary Results

S.3.1. Proof of Lemma 12

Proof Write matrix R as

$$R = \theta_{01}K_{01} + \theta_{11}K_{11} = \frac{1}{2}\begin{bmatrix} K_1^{\langle 1\rangle} & \theta_d K_1^{\langle 1\rangle} \\ \theta_d K_1^{\langle 1\rangle} & K_1^{\langle 1\rangle} \end{bmatrix},$$

where $\theta_d = \theta_{01} - \theta_{11}$. The inverse of M can be written as

$$\begin{aligned} M^{-1} &= \begin{bmatrix} \frac{1}{2}K_1^{\langle 1\rangle} + \lambda I_n & \frac{\theta_d}{2}K_1^{\langle 1\rangle} \\ \frac{\theta_d}{2}K_1^{\langle 1\rangle} & \frac{1}{2}K_1^{\langle 1\rangle} + \lambda I_n \end{bmatrix}^{-1} \triangleq \begin{bmatrix} A & B \\ B & A \end{bmatrix}^{-1} \\ &= \begin{bmatrix} A^{-1} + A^{-1}B(A - BA^{-1}B)^{-1}BA^{-1} & -A^{-1}B(A-BA^{-1}B)^{-1} \\ -A^{-1}B(A-BA^{-1}B)^{-1} & (A-BA^{-1}B)^{-1} \end{bmatrix}, \end{aligned}$$

where $A = \frac{1}{2}K_1^{\langle 1\rangle} + \lambda I_n$, $B = \frac{\theta_d}{2}K$, and I_n denotes the $n \times n$ identity matrix. Note that S is a $2n \times 2$ matrix defined as $S = (\mathbf{1}_n, \mathbf{1}_n)^T$. We thus have

$$S^T M^{-1} S = \begin{bmatrix} a & b \\ b & c \end{bmatrix},$$

where

$$\begin{aligned} a &= \mathbf{1}^T A^{-1}\mathbf{1} + \mathbf{1}^T A^{-1}B(A - BA^{-1}B)^{-1}BA^{-1}\mathbf{1} + 2b - c, \\ b &= -\mathbf{1}^T BA^{-1}(A - BA^{-1}B)^{-1}\mathbf{1} + \mathbf{1}^T(A - BA^{-1}B)^{-1}\mathbf{1}, \\ c &= \mathbf{1}^T(A - BA^{-1}B)^{-1}\mathbf{1}. \end{aligned}$$

Consequently,

$$\begin{aligned} S(S^TM^{-1}S)^{-1}S^T &= \frac{1}{ac-b^2}\begin{bmatrix} c\mathbf{1}\mathbf{1}^T & (c-b)\mathbf{1}\mathbf{1}^T \\ (c-b)\mathbf{1}\mathbf{1}^T & (a+c-2b)\mathbf{1}\mathbf{1}^T \end{bmatrix} \\ &= \frac{1}{ac-b^2}\begin{bmatrix} c\mathbf{1}\mathbf{1}^T & (c-b)\mathbf{1}\mathbf{1}^T \\ (c-b)\mathbf{1}\mathbf{1}^T & c\mathbf{1}\mathbf{1}^T \end{bmatrix}, \end{aligned}$$

where the second equality holds by the fact $a - 2b = 0$ and Woodbury matrix identity.

Note that $\boldsymbol{f}_{10} = (f_{10}(x_1^{\langle 1\rangle}), \ldots, f_{10}(x_n^{\langle 1\rangle}), f_{10}(x_1^{\langle 1\rangle}), \ldots, f_{10}(x_n^{\langle 1\rangle}))$. Let

$$(f_{10}(x_1^{\langle 1\rangle}), \ldots, f_{10}(x_n^{\langle 1\rangle}) \triangleq \mathbf{h}^T.$$

Therefore, we have

$$\begin{aligned} &K_{11}M^{-1}(I_n - S(S^TM^{-1}S)^{-1}S^TM^{-1})\boldsymbol{f}_{10} \\ =&\frac{1}{2}\begin{bmatrix} K_1^{\langle 1\rangle} & -K_1^{\langle 1\rangle} \\ -K_1^{\langle 1\rangle} & K_1^{\langle 1\rangle} \end{bmatrix}(M^{-1} - \frac{1}{ac-b^2}M^{-1}\begin{bmatrix} c\mathbf{1}\mathbf{1}^T & (c-b)\mathbf{1}\mathbf{1}^T \\ (c-b)\mathbf{1}\mathbf{1}^T & c\mathbf{1}\mathbf{1}^T \end{bmatrix}M^{-1})\begin{bmatrix} \mathbf{h} \\ \mathbf{h} \end{bmatrix}. \end{aligned}$$

Since both M^{-1} and $\frac{1}{ac-b^2}M^{-1}\begin{bmatrix} c\mathbf{1}\mathbf{1}^T & (c-b)\mathbf{1}\mathbf{1}^T \\ (c-b)\mathbf{1}\mathbf{1}^T & c\mathbf{1}\mathbf{1}^T \end{bmatrix} M^{-1})$ are symmetric matrices and their diagnonal entries are identical, we have

$$\begin{bmatrix} \mathbf{h}^* \\ \mathbf{h}^* \end{bmatrix} \triangleq (M^{-1} - \frac{1}{ac-b^2}M^{-1}\begin{bmatrix} c\mathbf{1}\mathbf{1}^T & (c-b)\mathbf{1}\mathbf{1}^T \\ (c-b)\mathbf{1}\mathbf{1}^T & c\mathbf{1}\mathbf{1}^T \end{bmatrix} M^{-1}) \begin{bmatrix} \mathbf{h} \\ \mathbf{h} \end{bmatrix}.$$

Simple algebra yields $K_{11}M^{-1}(I_n - S(S^TM^{-1}S)^{-1}S^TM^{-1})\boldsymbol{f}_{10} = 0$. ■

S.3.2. PROOF OF LEMMA 13

Proof Under the quasi-uniform design, $X_1^{\langle 1\rangle}$,..., $X_n^{\langle n\rangle}$ are i.i.d with distribution $\omega^{\langle 1\rangle}$. Therefore, by Theorem 3 in Braun (2006), for $1 \le i \le n$ and $i \le r \le n$, simple algebra yields

$$\mathbb{P}(|\hat{\mu}_i - \mu_i| \le c_m\mu_i + \mu_r + \Lambda_r) \ge 1 - r(r+1)\exp\{-\frac{nc_m^2}{2C^4r^2}\},$$

where $\Lambda_r = \sum_{i=r+1}^{\infty}\mu_i$, C is an absolute constant, and c_m is a constant depending solely on m. Since the eigenvalue μ_i has the polynomial decay rate i^{-2m}, we have

$$\Lambda_r \asymp \sum_{i=r+1}^{\infty} i^{-2m}.$$

For $m > 1/2$,

$$\sum_{i=r+1}^{\infty} i^{-2m} \le \int_r^{\infty} x^{-2m}dx = \frac{r^{1-2m}}{2m-1} = \mathcal{O}(r^{1-2m}).$$

Let $r = n^{1/(2m-1)-\epsilon}$, we have $\Lambda_r + \mu_r = \mathcal{O}(n^{2\epsilon m-1-\epsilon}) = o(\mu_i)$ for $i = 1, \ldots, n^{1/2m-\epsilon}$. Next, we have, for any $i = 1, \ldots, n^{\frac{1}{2m}-\epsilon}$, the empirical eigenvalue $\hat{\mu}_i$ satisfies

$$|\hat{\mu}_i - \mu_i| \le c_m\mu_i,$$

with probability at least

$$1 - (n^{\frac{2}{2m-1}-2\epsilon} + n^{\frac{1}{2m-1}})\exp\{-cn^{\frac{2m-3}{2m-1}+2\epsilon}\}, \tag{S.4}$$

where $c = \frac{c_m^2}{2C^4}$, c_m is a constant only related to m, and M is an absolute constant. To ensure the probability in Equation (S.4) goes to 1, we further require $m > 3/2$. Thus, for $\lambda > 1/n$ and $m > 3/2$,

$$\hat{\tau}_\lambda \asymp \tau_\lambda$$

with probability at least $1 - (n^{\frac{2}{2m-1}-2\epsilon} + n^{\frac{1}{2m-1}})\exp\{-cn^{\frac{2m-3}{2m-1}+2\epsilon}\}$. ■

S.3.3. PROOF OF LEMMA 14

Proof Considering $\mathcal{H}^{\langle 1\rangle}$ as the homogeneous Sobolev space, the kernel function $\mathcal{K}_1^{\langle 1\rangle}$ can be explicitly written as

$$\mathcal{K}_1^{\langle 1\rangle}(x,y) = 2\sum_{i=1}^{\infty} \frac{cos(2\pi k(x-y))}{(2\pi k)^{2m}}.$$

Under the uniform design, we have the $X_1^{\langle 1\rangle}, \ldots, X_n^{\langle 1\rangle}$ evenly distributed on $[0,1]$. Without loss of generality, we assume that $X_1^{\langle 1\rangle} < \cdots < X_n^{\langle 1\rangle}$. Therefore, the ii'th entry of kernel matrix $K_1^{\langle 1\rangle}$ is $\mathcal{K}_1^{\langle 1\rangle}(x_i^{\langle 1\rangle}, x_{i'}^{\langle 1\rangle})$ which is a symmetric circulant matrix of order n (Shang and Cheng, 2017) with eigenvalues

$$\hat{\mu}_i^* = \begin{cases} \sum_{k=1}^{\infty} \frac{1}{[2\pi(kn-i)]^{2m}} + \sum_{k=0}^{\infty} \frac{1}{[2\pi(kn+i)]^{2m}} & \text{if } 1 \le i \le n-1 \\ 2\sum_{k=1}^{\infty} \frac{1}{(2\pi kn)^{2m}} & \text{if } i = n \end{cases}. \tag{S.5}$$

Note that $\hat{\mu}_i^*$ is a re-arrangement of $\hat{\mu}_i$. When $m > 1/2$, simple calculation yields

$$\begin{aligned} \frac{1}{[2\pi(n-i)]^{2m}} + \frac{1}{(2\pi i)^{2m}} + 2\underline{c}_m(2\pi n)^{-2m} \le & \hat{\mu}_i^* \\ \le & \frac{1}{[2\pi(n-i)]^{2m}} + \frac{1}{(2\pi i)^{2m}} + 2\bar{c}_m(2\pi n)^{-2m}, \end{aligned} \tag{S.6}$$

for $i = 1, \ldots, n-1$, and

$$\hat{\mu}_n^* = 2\bar{c}_m(2\pi n)^{-2m},$$

where $\underline{c}_m := \sum_{k=1}^{\infty} k^{-2m}$, and $\bar{c}_m = \sum_{k=2}^{\infty} k^{-2m}$. By Equation (S.6), we have $\hat{\mu}_i^* \asymp \mu_i$ for $1 \le i \le \frac{n}{2}$ and $\hat{\mu}_i^* \asymp \mu_{n-i}$ for $\frac{n}{2} \le i \le n$. Since $\{\hat{\mu}\}_{i=1}^n$ are obtained by ordering $\{\hat{\mu}_i^*\}_{i=1}^n$ decreasingly, we have $\mu_i \asymp \hat{\mu}_i$, and consequently,

$$\tau_\lambda \asymp \hat{\tau}_\lambda$$

for any $\lambda > 0$. ■

S.3.4. PROOF OF LEMMA 15

Proof

Note that the kernel matrix $K_1^{\langle 1\rangle}$ in Equation (A.1) has the spectral decomposition $K_1^{\langle 1\rangle} = UDU^T$, where the eigenvector matrix U is a $n \times n$ unitary matrix and the eigenvalue matrix $D = Diag\{\hat{\mu}_i\}$ is a diagonal matrix with eigenvalues $\hat{\mu}_1 \ge \hat{\mu}_2 \ge \cdots \ge \hat{\mu}_n$. Correspondingly, we have the following decomposition,

$$K_{11} = \frac{1}{2}\begin{bmatrix} U & 0 \\ 0 & U \end{bmatrix} \begin{bmatrix} D & -D \\ -D & D \end{bmatrix} \begin{bmatrix} U^T & 0 \\ 0 & U^T \end{bmatrix},$$

$$M = \frac{1}{2}\begin{bmatrix} U & 0 \\ 0 & U \end{bmatrix} \begin{bmatrix} D + 2\lambda I_n & \theta_d D \\ \theta_d D & D + 2\lambda I_n \end{bmatrix} \begin{bmatrix} U^T & 0 \\ 0 & U^T \end{bmatrix},$$

where I_n is the $n \times n$ identity matrix, and $\theta_d = \theta_{01} - \theta_{11}$. Letting $E = D + 2\lambda I_n = Diag\{\hat{\mu}_i + 2\lambda\}$ and $F = \theta_d D = Diag\{\theta_d \hat{\mu}_i\}$, we have

$$K_{11} M^{-1} = \begin{bmatrix} U & 0 \\ 0 & U \end{bmatrix} \begin{bmatrix} D & -D \\ -D & D \end{bmatrix} \begin{bmatrix} E & F \\ F & E \end{bmatrix}^{-1} \begin{bmatrix} U^T & 0 \\ 0 & U^T \end{bmatrix}.$$

Using the inverse of block matrix, we have

$$\begin{bmatrix} D & -D \\ -D & D \end{bmatrix} \begin{bmatrix} E & F \\ F & E \end{bmatrix}^{-1} \triangleq \begin{bmatrix} V_{11} & V_{12} \\ V_{21} & V_{22} \end{bmatrix}$$

where

$$\begin{aligned} V_{11} &= DE^{-1} + (D + DE^{-1}F)(E - FE^{-1}F)^{-1} FE^{-1}, \\ V_{12} &= -(DE^{-1}F + D)(E - FE^{-1}F)^{-1}, \\ V_{21} &= -V_{12}, && \text{(S.7)} \\ V_{22} &= -V_{11}. && \text{(S.8)} \end{aligned}$$

Consequently,

$$\Delta = M^{-1} K_{11}^2 M^{-1} = \begin{bmatrix} U & 0 \\ 0 & U \end{bmatrix} \begin{bmatrix} V_{11} & V_{12} \\ V_{21} & V_{22} \end{bmatrix}^T \begin{bmatrix} V_{11} & V_{12} \\ V_{21} & V_{22} \end{bmatrix} \begin{bmatrix} U^T & 0 \\ 0 & U^T \end{bmatrix}.$$

We thus have

$$\begin{aligned} \mathrm{Tr}(\Delta) = \mathrm{Tr}(M^{-1} K_{11}^2 M^{-1}) &= \mathrm{Tr}(\begin{bmatrix} V_{11} & V_{12} \\ V_{21} & V_{22} \end{bmatrix}^T \begin{bmatrix} V_{11} & V_{12} \\ V_{21} & V_{22} \end{bmatrix}) \\ &= \mathrm{Tr} \begin{bmatrix} V_{11}^T V_{11} + V_{21}^T V_{21} & V_{11}^T V_{12} + V_{21}^T V_{22} \\ V_{12}^T V_{11} + V_{22}^T V_{21} & V_{12}^T V_{12} + V_{22}^T V_{22} \end{bmatrix}. \end{aligned} \tag{S.9}$$

By Equation (S.7) and Equation (S.8), we have

$$V_{11}^T V_{11} + V_{21}^T V_{21} = V_{12}^T V_{12} + V_{22}^T V_{22}.$$

Simple algebra yields

$$V_{11}^T V_{11} + V_{21}^T V_{21} = 2 V_{11}^T V_{11}.$$

Therefore, we have

$$\mathrm{Tr}(\Delta) = 4 \, \mathrm{Tr}(V_{11}^T V_{11}). \tag{S.10}$$

Notice that D, E, F are diagonal matrices, we have

$$\mathrm{Tr}(\Delta) = 4 \, \mathrm{Tr}(V_{11}^T V_{11}) \geq 4 \, \mathrm{Tr} \, (D^2 E^{-2}).$$

Since

$$D^2 E^{-2} = Diag\{\frac{\hat{\mu}_i^2}{(\hat{\mu}_i + 2\lambda)^2}\},$$

we have

$$\mathrm{Tr}(\Delta) \geq 4 \sum_{i=1}^{n} \frac{\hat{\mu}_i^2}{(\hat{\mu}_i + 2\lambda)^2} \geq 4 \sum_{i=1}^{\hat{r}_\lambda} \frac{\hat{\mu}_i^2}{(\hat{\mu}_i + 2\lambda)^2}, \tag{S.11}$$

where $\hat{\tau}_\lambda$ is the effective dimension for kernel matrix K_{11}. For the any $i < \hat{\tau}_\lambda$, we have $\frac{\hat{\mu}_i}{\hat{\mu}_i+2\lambda} > \frac{1}{3}$. Thus we have

$$\mathrm{Tr}(\Delta) \geq \frac{4}{9}\hat{\tau}_\lambda.$$

Now we shall prove the upper bound for $\mathrm{Tr}(\Delta)$. Since $\mathrm{Tr}(\Delta)$ has the expression in Equation (S.10), we expand V_{11} as

$$V_{11} = DE^{-1} + DE^{-1}(F(E - FE^{-1}F)^{-1}FE^{-1} + (E - FE^{-1})^{-1}F).$$

The ith diagonal entry of $F(E - FE^{-1}F)^{-1}FE^{-1}$ is

$$\begin{aligned} Diag_i(F(E - FE^{-1}F)^{-1}FE^{-1}) &= \frac{\theta_d^2\hat{\mu}_i^2}{(\hat{\mu}_i + 2\lambda - \frac{\theta_d^2\hat{\mu}_i^2}{\hat{\mu}_i+2\lambda})(\hat{\mu}_i + 2\lambda)} \\ &\leq \frac{\theta_d^2}{1-\theta_d^2}, \end{aligned} \tag{S.12}$$

and the ith diagonal entry of $(E - FE^{-1})^{-1}F$ is

$$Diag_i((E - FE^{-1})^{-1}F) = \frac{\theta_d\hat{\mu}}{\hat{\mu}_i + 2\lambda - \frac{\theta_d^2\hat{\mu}_i^2}{\hat{\mu}_i+2\lambda}} \leq \frac{\theta_d}{1-\theta_d^2}. \tag{S.13}$$

Combining Equation (S.12) and Equation (S.13), we have the ith diagonal entry of V_{11}

$$Diag_i(V_{11}) \leq (1 + \frac{\theta_d^2}{1-\theta_d^2} + \frac{\theta_d}{1-\theta_d^2}Diag_i(DE^{-1}) = \frac{1}{1-\theta_d}Diag_i(DE^{-1}).$$

Since the lower diagonal block of DE^{-1} is identical to the upper diagonal block, we only need to bound the trace of DE^{-1}. We have

$$\begin{aligned} \mathrm{Tr}(D^2E^{-2}) =& \sum_{i=1}^{\hat{\tau}_\lambda} \frac{\hat{\mu}_i^2}{(\hat{\mu}_i + 2\lambda)^2} + \sum_{i=\hat{\tau}_\lambda+1}^{n} \frac{\hat{\mu}_i^2}{(\hat{\mu}_i + 2\lambda)^2} \\ \leq& \sum_{i=1}^{\hat{\tau}_\lambda} \frac{\hat{\mu}_i}{\hat{\mu}_i + 2\lambda} + \sum_{i=\hat{\tau}_\lambda+1}^{n} \frac{\hat{\mu}_i}{\hat{\mu}_i + 2\lambda} \\ \leq& \hat{\tau}_\lambda + \frac{1}{2\lambda}\sum_{i=\hat{\tau}_\lambda+1}^{n} \hat{\mu}_i. \end{aligned}$$

Thus we have $\mathrm{Tr}(\Delta) \leq \frac{4}{(1-\theta_d)^2}(\hat{\tau}_\lambda + \frac{1}{2\lambda}\sum_{i=\hat{\tau}_\lambda+1}^{n} \hat{\mu}_i)$. ■

S.3.5. Proof of Lemma S.1

Proof By the functional decomposition in Equation (10), we have

$$||f_{10} + f_{11}||^2_{\mathcal{H}_{10}\oplus\mathcal{H}_{11}} \leq ||f||^2_{\mathcal{H}} < 1. \tag{S.14}$$

For any function g in $\mathcal{H}_{10} \oplus \mathcal{H}_{11}$, we write $g = \xi^T \tilde{\mathbf{c}} + \zeta(\cdot)$, where $\zeta(\cdot) \in \mathcal{H}_{10} \oplus \mathcal{H}_{11}$ is orthogonal to ξ. Moreover,

$$\begin{aligned} ||f_{10} + f_{11}||^2_{\mathcal{H}_{10}\oplus\mathcal{H}_{11}} =& ||\xi^T \tilde{\mathbf{c}}||^2_{\mathcal{H}_{10}\oplus\mathcal{H}_{11}} + ||\zeta(\cdot)||^2_{\mathcal{H}_{10}\oplus\mathcal{H}_{11}} \\ \geq & n\tilde{\mathbf{c}}^T R \tilde{\mathbf{c}} = \frac{1}{n}(n\tilde{\mathbf{c}}^T R)R^{-1}(nR\tilde{\mathbf{c}}) \\ =& \frac{1}{n}\boldsymbol{g}^{*T} R^{-1} \boldsymbol{g}^*. \end{aligned} \tag{S.15}$$

Combining Equation (S.14) and Equation (S.15), we have

$$\frac{1}{n}\boldsymbol{g}^{*T} R^{-1} \boldsymbol{g}^* < 1. \tag{S.16}$$

By Equation (S.1), we have

$$\begin{aligned} ||\tilde{g}^* - g^*||^2_n = & \frac{1}{n}||\boldsymbol{g}^* - RM^{-1}\boldsymbol{g}^* + RM^{-1}S(S^T M^{-1} S)^{-1} S^T M^{-1} \boldsymbol{g}^*||^2_2 \\ &= \frac{1}{n}\boldsymbol{g}^{*T}(I - RM^{-1})^2 \boldsymbol{g}^* + \frac{1}{n}||RM^{-1}S(S^T M^{-1} S^T)^{-1} S^T M^{-1} \boldsymbol{g}^*||^2_2. \end{aligned}$$

Noting that $M = R + \lambda I_n$, the eigenvalues of $I_n - R(R + \lambda I_n)^{-1}$ are all smaller than 1, and the rank of $RM^{-1}S(S^T M^{-1} S^T)^{-1} S^T M^{-1}$ is 2, we have

$$\begin{aligned} ||\tilde{g}^* - g^*||^2_n \leq & \frac{1}{n}\boldsymbol{g}^*(I - R(R + \lambda I)^{-1})\boldsymbol{g}^* + \mathcal{O}(\frac{1}{n}) \\ \leq & \lambda + \mathcal{O}(\frac{1}{n}), \end{aligned}$$

where the last inequality holds by applying Woodbury matrix identity,

$$(R + \lambda I_n)^{-1} = R^{-1} - R^{-1}(\frac{1}{\lambda} I_n + R^{-1})^{-1} R^{-1} \geq R^{-1} - \lambda R^{-2},$$

and Equation (S.16). The proof is thus completed. ■

S.3.6. PROOF OF LEMMA S.2

Proof Under the quasi-uniform design, Theorem 3.1 of Eggermont and LaRiccia (2001)(page 384, Eggermont and LaRiccia (2001)) implies that $||\cdot||_{\omega^{\langle 1\rangle} mh}$ norm is equivalent to $||\cdot||_n$ for any fixed $x^{\langle 2\rangle}$. The $||\cdot||_{\omega^{\langle 1\rangle} hm}$, which is defined as $||f(x^{\langle 1\rangle}, 0)||^2_{\omega^{\langle 1\rangle} mh} = ||f(x^{\langle 1\rangle}, 0)||^2_{L_2(\omega^{\langle 1\rangle})} + h^{2m}||f(x^{\langle 1\rangle}, 0)^{(m)}||^2$, trivially dominates the $||\cdot||_{L_2(\omega^{\langle 1\rangle})}$ norm. Since $x^{\langle 2\rangle}$ can only take the values 0 or 1, we have that $||\cdot||_n$ dominates $||\cdot||_2$, i.e. there exists a positive constant c such that $||f||_2 \leq c||f||_n$.

Under the uniform design, Lemma 2.27 in Eggermont and LaRiccia (2001) states that $||\cdot||_n$ dominates $||\cdot||_2$ for $x^{\langle 1\rangle}$.

■

S.3.7. PROOF OF LEMMA S.4

Proof Under the uniform design, the empirical eigenvalues could be calculated by Equation (S.5). By the definition of $\hat{\tau}_\lambda$, we have

$$\sum_{i=\hat{\tau}_\lambda+1}^{n} \hat{\mu}_i = \sum_{\{i|\hat{\mu}_i^*<\lambda\}} \hat{\mu}_i^*. \tag{S.17}$$

Since the population eigenvalues are $\{(2\pi i)^{-2m}\}_{i=1}^{\infty}$, we calculate the population efficient dimension as $\tau_\lambda = (\lambda)^{-1/2m}/2\pi$. By the inequalities Equation (S.6), we have $\hat{\mu}_i^* \geq \lambda$ for $i = 1, \ldots, \tau_\lambda$ or $i = n - \tau_\lambda, \ldots, n$. We can bound the term in Equation (S.17)

$$\sum_{\{i|\hat{\mu}_i^*<\lambda\}} \hat{\mu}_i^* \leq \sum_{i=\tau_\lambda}^{n-\tau_\lambda} \hat{\mu}_i^*.$$

By the upper bound of $\hat{\mu}_i^*$ given in Equation (S.6), we have

$$\sum_{i=\tau_\lambda}^{n-\tau_\lambda} \hat{\mu}_i^* \leq C\tau_\lambda \mu_{\tau_\lambda},$$

which completes the proof. ∎
